# Paediatric, maternal, and congenital mpox: a systematic review and meta-analysis

**DOI:** 10.1016/S2214-109X(23)00607-1

**Published:** 2024-02-21

**Authors:** Nuria Sanchez Clemente, Charlotte Coles, Enny S Paixao, Elizabeth B Brickley, Elizabeth Whittaker, Tobias Alfven, Stephen Rulisa, Nelson Agudelo Higuita, Paul Torpiano, Priyesh Agravat, Emma V Thorley, Simon B Drysdale, Kirsty Le Doare, Jean-Jacques Muyembe Tamfum

**Affiliations:** a Centre for Neonatal and Paediatric Infection, St George's University, London, UK; b Health Equity Action Lab, Department of Infectious Disease Epidemiology, London School of Hygiene & Tropical Medicine, London, UK; c Paediatric Infectious Diseases, Imperial College Healthcare NHS Trust, London, UK; d Section of Paediatric Infectious Diseases, Imperial College London, London, UK; e Centre of Excellence in Maternal Vaccination, Makerere University, John Hopkins University, Kampala, Uganda; f Pathogen Immunology Group, UK Health Security Agency, Porton Down, UK; g Department of Global Public Health, Karolinska Institutet, Stockholm, Sweden; h Sachs' Children and Youth Hospital, Stockholm, Sweden; i School of Medicine and Pharmacy, University of Rwanda and University Teaching Hospital of Kigali, Kigali, Rwanda; j Department of Medicine, Section of Infectious Diseases, University of Oklahoma Health Sciences Center, Oklahoma City, OK, USA; k Instituto de Enfermedades Infecciosas y Parasitología Antonio Vidal, Tegucigalpa, Honduras; l Department of Paediatrics and Adolescent Health, Mater Dei Hospital, Malta; m Faculty of Medicine, University of Kinshasa, Kinshasa, Democratic Republic of the Congo

## Abstract

**Background:**

Although mpox has been detected in paediatric populations in central and west Africa for decades, evidence synthesis on paediatric, maternal, and congenital mpox, and the use of vaccines and therapeutics in these groups, is lacking. A systematic review is therefore indicated to set the research agenda.

**Methods:**

We conducted a systematic review and meta-analysis, searching articles in Embase, Global Health, MEDLINE, CINAHL, Web of Science, Scopus, SciELO, and WHO databases from inception to April 17, 2023. We included studies reporting primary data on at least one case of confirmed, suspected, or probable paediatric, maternal, or congenital mpox in humans or the use of third-generation smallpox or mpox vaccines, targeted antivirals, or immune therapies in at least one case in our population of interest. We included clinical trials and observational studies in humans and excluded reviews, commentaries, and grey literature. A pooled estimate of the paediatric case fatality ratio was obtained using random-effects meta-analysis. This study is registered with PROSPERO (CRD420223336648).

**Findings:**

Of the 61 studies, 53 reported paediatric outcomes (n=2123 cases), seven reported maternal or congenital outcomes (n=32 cases), two reported vaccine safety (n=28 recipients), and three reported transmission during breastfeeding (n=4 cases). While a subset of seven observational studies (21 children and 12 pregnant individuals) reported uneventful treatment with tecovirimat, there were no randomised trials reporting safety or efficacy for any therapeutic agent. Among children, the commonest clinical features included rash (86 [100%] of 86), fever (63 [73%] of 86), and lymphadenopathy (40 [47%] of 86). Among pregnant individuals, rash was reported in 23 (100%) of 23; fever and lymphadenopathy were less common (six [26%] and three [13%] of 23, respectively). Most paediatric complications (12 [60%] of 20) arose from secondary bacterial infections. The pooled paediatric case fatality ratio was 11% (95% CI 4–20), *I*^2^=75%. Data from 12 pregnancies showed half resulted in fetal death. Research on vaccine and immune globulin safety remains scarce for children and absent for pregnant individuals.

**Interpretation:**

Our review highlights critical knowledge gaps in the epidemiology, prevention, and treatment of mpox in children and pregnant individuals, especially those residing in endemic countries. Increased funding, international collaboration, and equitable research is needed to inform mpox control strategies tailored for at-risk communities in endemic countries.

**Funding:**

None.

**Translations:**

For the French, Spanish and Portuguese translations of the abstract see Supplementary Materials section.

## Introduction

Mpox (previously monkeypox) is a zoonotic orthopox viral disease that has historically affected infants, children, and adolescents from lower-income and rural communities in west and central Africa. The true burden and geographical range of endemic mpox remains poorly defined due to diagnostic challenges and surveillance limitations.^1^ However, the incidence is typically highest in children, with available recent data from DR Congo indicating an estimated incidence of 18·1 per 100 000 among 5–9-year-olds.^2^

The primary reservoir in west and central Africa is thought to be small mammals (eg, rope squirrels, giant-pouched rats, and African dormice), although live monkeypox virus (MPXV) has only been isolated from sylvatic animals twice.^3^, ^4^ Primates can also become infected, and the hunting, handling, and consumption of bushmeat has been implicated in mpox cases.^5^, ^6^, ^7^ Human-to-human transmission occurs via direct contact with lesion exudates, bodily fluids, or respiratory droplets, and indirect contact with contaminated surfaces.^1^ Mpox can also be vertically transmitted^8^, ^9^, ^10^ and has the potential to be transmitted via breastfeeding.^11^, ^12^, ^13^

In 2022, a multi-country mpox outbreak occurred,^14^, ^15^, ^16^, ^17^ primarily due to viral clade IIb (which is less virulent than clade I), resulting in 91 123 cases across 115 countries and 157 deaths, as of October, 2023, and triggering WHO to declare a Public Health Emergency of International Concern.^18^, ^19^ Despite the recognition of human cases since 1970 and the high number of cases in this outbreak, research on paediatric and pregnant populations remains scarce.^20^ Children are at particular risk of severe sequelae, including keratitis leading to blindness,^9^ severe keloid scarring, pneumonitis, and encephalitis.^21^, ^22^, ^23^

**Research in context**

**Evidence before this study**

Mpox, caused by the monkeypox virus, has been documented to affect rural communities in central and west Africa since the 1970s. The incidence is highest among infants, children, and adolescents in these regions and pregnant women are at risk of severe complications including fetal loss. Before the multi-country outbreak in 2022, mpox was an extremely neglected disease, but despite the increasing interest, research on the sequelae, treatment, and prevention of mpox in children and pregnant women remains limited. Before this study, we did a rapid review on PubMed on June 1, 2022, for studies on paediatric and maternal mpox, using the search terms monkeypox or mpox, pregnan*, maternal, wom#n, child*, congenital*, vertical*, transmi*, neonat*, p?ediatric, infect*, smallpox, vaccin* or immuni#ation, prevent*, safe*, tecovirimat, treat*, and breastfeeding. A systematic review on the epidemiology of mpox reported an increase in the incidence of mpox in all age groups in DR Congo and an increase in the median age of published cases. A systematic review on maternal mpox based on four pregnant individuals (data to June, 2022) reported an incidence of late fetal and perinatal loss of 77·0% (95% CI 26·0−100). There were no systematic reviews reporting on paediatric mpox.

**Added value of this study**

This is the first systematic review to collate clinical and therapeutic evidence on paediatric and maternal mpox in both endemic and non-endemic countries. Our systematic review includes clinical data from 16 countries on 2123 paediatric cases and 32 maternal cases. It provides evidence of third-generation smallpox vaccination safety in 28 paediatric cases, uneventful tecovirimat use in 21 paediatric and 12 maternal cases, vaccinia immune globulin intravenous use in four paediatric cases, and cidofovir use in one paediatric case. It provides evidence of poor outcomes (fetal death) in half (six of 12) of the cases who were infected during pregnancy (occurring in all three trimesters). It provides a pooled estimate of the case fatality ratio of mpox in children of 11% (95% CI 4–20). The scope of the results is limited by the availability and quality of the datasets that have been published.

**Implications of all the available evidence**

Mpox can lead to devastating sequelae for children and pregnant women in endemic regions in Africa. There are currently no completed randomised controlled trials investigating the effectiveness of treatments in pregnant and paediatric populations, there is scant access to appropriate diagnostic methods, and little if any availability of the third-generation vaccine in endemic regions in Africa. Funding and priority should be given to the surveillance of mpox and the collection of disaggregated longitudinal data in endemic regions. This should be supported by the rapid scaling up of free testing with government policy backing to maximise the capture of cases. Adult, paediatric, and pregnant populations in endemic countries must also be included in clinical trials assessing the efficacy of novel treatments and vaccines. This includes appropriate vaccine allocation and distribution prioritising the most at-risk groups. Increased funding, international collaboration, and equitable research is ultimately needed to contain this neglected disease beginning with those most at risk in endemic countries.

The risks associated with mpox can be reduced through vaccine administration and therapeutics. A live, attenuated, non-replicating third-generation smallpox vaccine (MVA-BN available as JYNNEOS or IMVANEX) was licensed against smallpox by the European Medicines Agency in 2013 and extended in 2020 to include mpox and related orthopoxviruses (OPXV). It is currently unlicensed in children younger than 18 years,^24^, ^25^ although emergency use authorisation is available for children in some high-income and middle-income regions, including the EU, UK, and USA.^26^ Its use in pregnancy has not been published but has demonstrated safety in animal models.^27^ Another third-generation vaccine, LC16-KMB, is licensed in children as part of a clinical trial in Japan.^28^ The first-generation Dryvax and second-generation ACAM2000 vaccines had a vaccine effectiveness of 72% and 75% respectively,^29^ but these and other older smallpox vaccines were considered unsafe in pregnancy and contraindicated in immunosuppressed individuals and infants.^30^, ^31^, ^32^, ^33^, ^34^ Specific antiviral mpox treatments (eg, tecovirimat, brincidofovir) and immune therapies (eg, vaccinia immune globulin intravenous [VIGIV]) were used during the 2022 outbreak, but their use in children and pregnant women has been more limited.^16^, ^26^, ^35^, ^36^

To collate and appraise the available evidence, we did a systematic review on paediatric, maternal, and congenital mpox, vertical transmission, and the use of vaccines and therapeutics in pregnant and paediatric populations.

## Methods

### Search strategy and selection criteria

We conducted a systematic literature review in line with PRISMA guidelines,^37^ registering the study on PROSPERO (CRD420223336648). We employed a PICOS framework to structure the research (appendix 4 p 1).

We searched Embase, Global Health, MEDLINE, CINAHL, Web of Science, Scopus, SciELO, and WHO databases for primary research reporting on paediatric, maternal, and congenital mpox and the use of smallpox or mpox vaccines and treatments in our populations of interest, with no language, country, or date restrictions. A Boolean strategy was developed (appendix 4 pp 7–12). The initial search was carried out on June 17, 2022, and repeated on April 17, 2023. Ethical approval was not required.

We included studies reporting primary data on at least one case of confirmed, suspected, or probable paediatric, maternal, or congenital mpox in humans or the use of third-generation smallpox or mpox vaccines, targeted antivirals, or immune therapies in at least one case in our population of interest. We excluded review papers, commentaries, non-systematic reviews, and grey literature. Patients were considered paediatric if they were aged 18 years or younger. Adapted WHO 2022 mpox case definitions were used (appendix 4 pp 2–3).^38^ Cases of varicella zoster virus co-infection were excluded. For studies reporting on multiple populations or outcomes of interest, data were tabulated separately. Where multiple studies reported on the same cases or outbreaks, the most complete description was included.

### Data analysis

Records were imported into EndNote version 21, and duplicates deleted. One reviewer (NSC) carried out title and abstract screening, full-text screening, data extraction, and quality assessment, with all steps duplicated by an independent second reviewer (CC, PT, EVT, PA, or ESP). Discrepancies were resolved with a third researcher (PT or CC). We used a snowballing method to identify relevant articles through citations.

Data were extracted on study type, location, year, number of cases, and age and sex of the child or gestational age in weeks, plus symptoms, complications, and outcome of infection (recovered or died), diagnostic method (viral isolation, PCR, serology, or clinical diagnosis), and treatment.

Paediatric primary outcomes were complications secondary to mpox, requirement for hospitalisation or intensive care, and death. Maternal primary outcomes were preterm labour, pregnancy loss, requirement for hospitalisation or intensive care, and maternal death. Congenital primary outcomes were occurrence of mother-to-child mpox transmission, prematurity, small-for-gestational age, low birthweight, microcephaly, and congenital anomalies. Secondary outcomes were the safety and efficacy of smallpox or mpox vaccines and targeted antivirals or immune therapies in the prevention and treatment of mpox in pregnancy or childhood.

To estimate the pooled paediatric case fatality ratio (CFR; appendix 4 p 3), we conducted a meta-analysis using the random-effects model available from Stata version 18.0 metaprop function.^39^ This enabled the calculation of 95% CIs using the statistical score and the exact binomial method and incorporates the Freeman-Tukey arcsine double proportions transformation. This method also models intrastudy variability using the binomial distribution. Interstudy heterogeneity was described using the *I*^2^ statistic, which describes the percentage of variation across studies that is due to heterogeneity rather than chance. Where observational studies contained data from overlapping locations and time periods, studies with the greatest number of individuals were included and others were excluded. 95% CIs for proportions were also calculated using Stata version 18.0.^39^

Quality assessment of all studies was carried out in duplicate (NSC and CC, PT, EVT, PA, or ESP) using the Murad criteria, which categorise studies as good, fair, or poor.^40^

### Role of the funding source

There was no funding source for this study.

## Results

**Figure 1:**
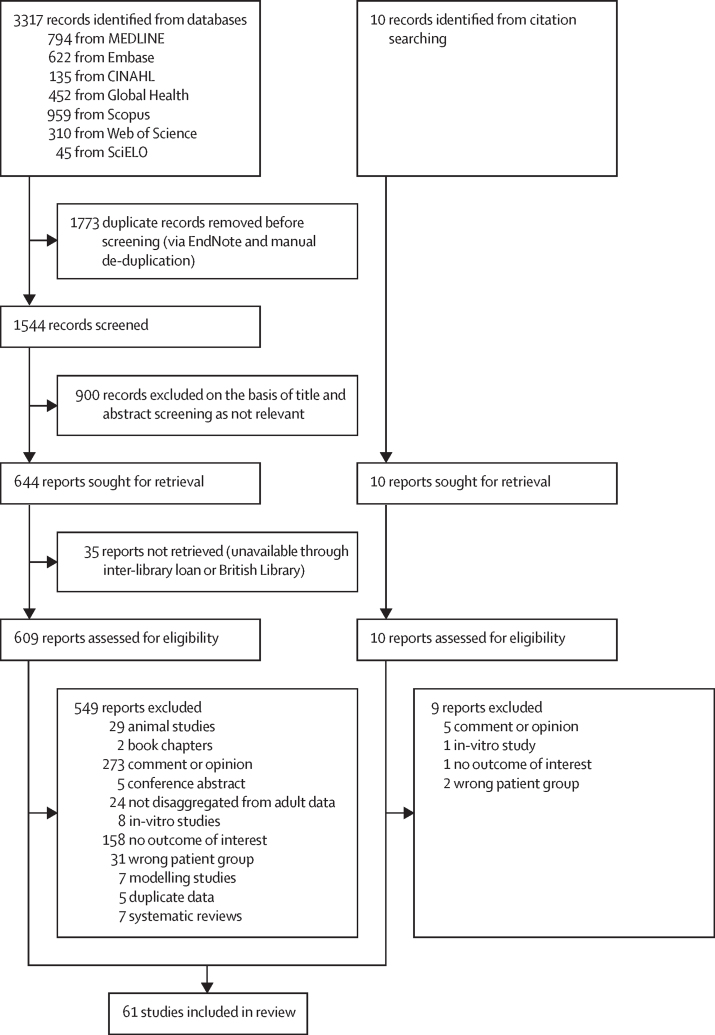
PRISMA flow diagram of included data sources

**Figure 2:**
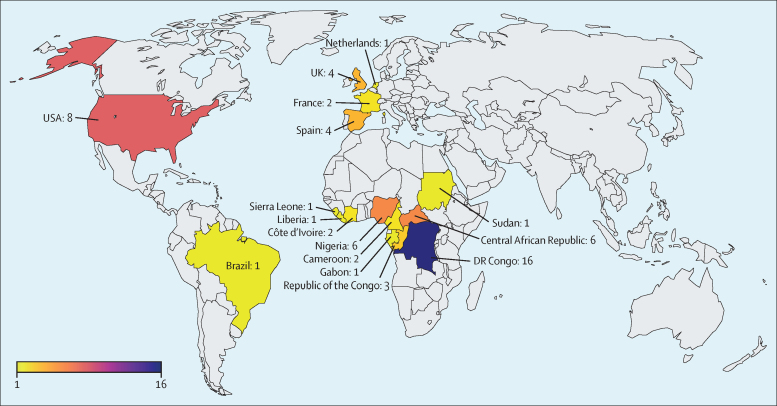
Map showing locations of included studies

Our database search identified 1544 records, with an additional ten identified via citation searching. Of these, 61 were included in the final analysis (figure 1), reporting on: paediatric outcomes (n=53), maternal or congenital outcomes (n=7), transmission through breastfeeding (n=3), and vaccination in pregnancy or paediatric populations (n=2), with four studies reporting on two domains. The studies reported cases from 16 countries (figure 2): DR Congo (n=16),^2^, ^5^, ^7^, ^8^, ^9^, ^23^, ^41^, ^42^, ^43^, ^44^, ^45^, ^46^, ^47^, ^48^, ^49^, ^50^, ^51^ USA (n=8),^11^, ^52^, ^53^, ^54^, ^55^, ^56^, ^57^, ^58^ Central African Republic (n=6),^1^, ^12^, ^59^, ^60^, ^61^, ^62^ Nigeria (n=6),^21^, ^63^, ^64^, ^65^ Spain (n=4),^66^, ^67^, ^68^ UK (n=4),^16^, ^69^, ^70^, ^71^ Republic of the Congo (n=3),^72^, ^73^, ^74^ Cameroon (n=2),^75^, ^76^ Côte d'Ivoire (n=2),^77^, ^78^ France (n=2),^79^, ^80^ Brazil (n=1),^81^ Gabon (n=1),^82^ Liberia (n=1),^83^ the Netherlands (n=1),^84^ Sierra Leone (n=1),^85^ and Sudan (pre-2011 split; n=1).^86^ One study reported Europe-wide data,^87^ and another reported data from west and central Africa.^88^ Publication dates spanned 51 years (1972–2023). Study designs included 18 case reports, 16 outbreak reports, ten observational studies, nine surveillance studies, seven case series, and one serological survey. Literature ranged in quality, with 30 studies rated as good, 28 as fair, and three as poor.^40^ Study characteristics are summarised in table 1, table 2, and appendix 4 (p 7), and quality assessments in appendix 4 (pp 4–5).

**Table 1:** Details of all included paediatric studies

		**Clinical features**	**Complications**	**Outcome**	**Laboratory diagnosis**	**Treatment**
**Non-aggregate data**						
Ditta et al (2023)^52^; case report; USA; n=1						
	8-year-old female	Right eye: pain, photophobia, blurry vision, discharge, multiple umbilicated lesions along eyelids, mild conjunctival injection	Corneal subepithelial and stromal infiltrates, preseptal cellulitis	Recovered; normal vision	PCR-positive (×2)	IV aciclovir (stopped); IV clindamycin; oral tecovirimat 400 mg 12-hourly for 14 days; trifluridine drops (keratitis)
Antonello et al (2023)^81^; case report; Brazil; n=1						
	9-day-old, sex NR	Generalised vesicular rash	Respiratory distress syndrome needing ventilation at day 11 of life	Recovered	PCR-positive (lesion)	IV vancomycin
Del Giudice et al (2023)^79^; case series; France; n=2						
	4-year-old female	Fever (38° C), rash (pustules, papules on erythematous base, disseminated erythematous maculae), bilateral conjunctivitis	Nil	Recovered	PCR-positive	Nil
	7-year-old female	Micropapular pustules on erythematous base (10 pustules)	Nil	Recovered	PCR-positive	Nil
Vallée et al (2023)^80^; case report; France; n=1						
	18-year-old female	Fever; rash on hands, wrists, gluteal region; ulceronecrotic lesions (vaginal and vulval); myalgia; headaches	Nil	Recovered	PCR-positive (throat swab)	Nil
Roguera Sopena et al (2022)^66^; case report; Spain; n=1						
	3-year-old female	Rash (polymorphous with umbilicated pustular, vesicular, papular elements)	Nil	Recovered	PCR-positive	NR
Fuente et al (2022)^67^; case report; Spain; n=1						
	13-month-old male	Fever; rash (purulent blistering lesion on finger and crusty lesions on scalp and toe); vomiting; diarrhoea	Nil	Recovered	PCR-positive	NR
Tutu van Furth et al (2022)^84^; case report; Netherlands; n=1						
	10-year-old male	Rash (20 solitary, sharply demarcated, red-brown vesicles); sore throat	NR	Recovered	PCR-positive (clade IIb)	Nil
Ramnarayan et al (2022)^69^; case report; UK; n=1						
	9-day-old, sex NR	Rash (initially vesicular then pustular); axillary lymphadenopathy	Hypoxaemic respiratory failure requiring ventilation; adenovirus co-infection	Recovered	PCR-positive (lesion fluid, blood, urine, respiratory secretions; clade IIb)	Enteral tecovirimat (2 weeks, 50 mg twice daily) and IV cidofovir
Saunders et al (2022)^53^; case report; USA; n=1						
	<2-month-old, sex NR	Raised erythematous rash on the arms, legs, and trunk	Cellulitis	Recovered	PCR-positive (lesion; clade II)	Oral tecovirimat and VIGIV
Adler et al (2022)^16^; case report; UK; n=1						
	<2-year-old female	No prodrome; lymphadenopathy; 30 concurrent lesions on face, trunk, arms, legs	NR	Recovered	PCR-positive (blood, nose and throat swab)	Nil*
Hobson et al (2021)^70^; case series; UK; n=1						
	18-month-old, sex NR	Rash	NR	Recovered	PCR-positive (lesion; clade II)	Nil
Ogoina et al (2020)^21^; retrospective observational study; Nigeria; n=1						
	28-day-old female	NR	Bronchopneumonia, lung opacification, encephalitis with seizures	Died	NR (diagnosis was reported as suspected, probable, or confirmed in an aggregate manner)	NR
Eltvedt et al (2020)^41^; case series; DR Congo; n=2						
	4-year-old male	Fever; rhinitis; conjunctivitis; cough; lymphadenitis; non-pruritic vesiculopapular rash (all skin surfaces including palms, soles, mucous membranes)	NR	Died	Nil (suspected case)	IV antibiotics, retinol, IV fluids, nutrition
	10-year-old male	NR	NR	Recovered	Nil (suspected case)	NR
Yinka-Ogunleye et al (2019)^64^; surveillance study; Nigeria; n=1						
	1-month-old, sex NR	Vesiculopapular rash	NR	Died	PCR-positive, IgM-positive (clade II)	NR
Sadeuh-Mba et al (2019)^75^; outbreak report; Cameroon; n=1						
	3-week-old female	Fever; generally unwell; painful maculopapular rash	NR	Recovered	PCR-negative (suspected)	NR
Reynolds et al (2019)^85^; outbreak report; Sierra Leone; n=1						
	11-month-old male	Fever; pustular umbilicated pruritic rash (all skin surfaces including palms, soles, mucous membranes); vomiting; loss of appetite; cough	Nil	Recovered	PCR-positive (serum, lesion); orthopoxvirus IgG and IgM-positive (clade II)	Nil
Ogoina et al (2019)^63^; outbreak report; Nigeria; n=1						
	11-year-old male	Fever; progressive vesicopustular rash on skin, oral, and nasal mucosa; generalised lymphadenopathy	NR	Recovered	NR (suspected)	NR
Doshi et al (2019)^72^; outbreak report; Republic of the Congo; n=14						
	9-year-old male	Fever, rash	NR	NR	PCR-positive (lesion)	NR
	4-year-old female	NR	NR	Died	PCR-positive (lesion)	NR
	12-year-old female	NR	NR	Recovered	Orthopoxvirus PCR-positive	NR
	8-year-old male	Rash	NR	NR	IgM, IgG-positive	NR
	14-year-old female	Fever, rash	NR	Died	Nil (suspected)	NR
	11-year-old female	Fever, rash	NR	Recovered	Orthopoxvirus PCR-positive	NR
	3-year-old male	Fever, rash	Nil	Recovered	IgM, IgG-positive	NR
	1-year-old female	NR	Nil	Recovered	IgG-positive	NR
	1-year-old male	NR	NR	Recovered	IgM, IgG-positive	NR
	5-year-old female	NR	NR	Recovered	IgM, IgG-positive	NR
	5-year-old female	NR	NR	Recovered	IgM, IgG-positive	NR
	3-year-old female	NR	NR	Recovered	IgM, IgG-positive	NR
	11-year-old male	NR	NR	Recovered	IgM, IgG-positive	NR
	15-year-old female	NR	NR	Recovered	IgG-positive	NR
Besombes et al (2019)^59^; outbreak report; Central African Republic; n=4						
	5-month-old female	Fever, maculopapular rash on soles and feet	NR	NR	PCR-positive	NR
	4-year-old female	Fever, maculopapular rash on soles and feet	NR	NR	PCR-positive	NR
	7-year-old female	Fever, rash	NR	NR	PCR-positive	NR
	16-year-old female	Fever, rash	NR	NR	PCR-positive	NR
Kalthan et al (2018)^60^; outbreak report; Central African Republic; n=1						
	1-year-old, sex NR	Fever, rash	NR	Died	NR (suspected)	NR
Nakoune et al (2017)^12^; outbreak report; Central African Republic; n=3						
	9-year-old male	Fever (which persisted for >7 days), rash, headaches	NR	Recovered	PCR-positive (blood)	Oral antibiotics
	5-year-old male	Fever, rash, cervical lymphadenitis, severe facial oedema, bilateral conjunctivitis	Pulmonary oedema, profound hypothermia	Died	PCR-positive (blood; clade I)	IV antibiotics, tetracycline eye ointment, furosemide, oxygen, promethazine
	15-month-old, sex NR	Rash, reduced feeding, lethargy	NR	Died	PCR-positive (blood)	Promethazine
Reynolds et al (2013)^73^; outbreak report; Republic of the Congo; n=6						
	2-year-old male	Fever, rash following monkey bite	NR	NR	PCR-negative	NR
	7-year-old female	NR	NR	NR	PCR-positive	NR
	16-year-old female	Fever, rash, lymphadenopathy	NR	NR	PCR-positive	NR
	10-year-old male	Fever, rash, lymphadenopathy	NR	NR	NR (suspected)	NR
	9-year-old female	Fever, rash, lymphadenopathy	NR	NR	NA (suspected)	NR
	12-year-old male	Fever, rash, lymphadenopathy	NR	NR	NA (suspected)	NR
Formenty et al (2010)^86^; outbreak report; Sudan; n=1						
	8-month-old male	Fever, rash, cough, lymphadenopathy	NR	Recovered	PCR-positive (blood and lesion; clade I)	NR
Learned et al (2005)^74^; case series; Republic of the Congo; n=11						
	16-year-old female	Rash	NR	Recovered	PCR-negative (suspected)	Flucloxacillin
	5-month-old female	Fever, rash, parotitis, dysphagia, lymphadenopathy	Became meningitic on day 8	Recovered	PCR-positive	Isoprinosine, Maxilase, ibuprofen, amoxicillin and gentamicin, ceftriaxone
	5-year-old male	Fever, rash, parotitis, dysphagia, lymphadenopathy	NR	Recovered	PCR-negative	Isoprinosine, Maxilase, ibuprofen, betamethasone, co-amoxiclav, gentamicin
	10-year-old male	Fever, rash, parotitis, dysphagia, lymphadenopathy	NR	Recovered	PCR-negative	Indomethacin, betamethasone, benzylpenicillin, gentamicin
	10-year-old female	Fever, rash, dysphagia, lymphadenopathy, conjunctivitis	Developed renal tract sepsis, had surgical exploration	Died	PCR-positive	Surgery
	8-year-old female	Fever, rash, dysphagia, lymphadenopathy, conjunctivitis	NR	Recovered	PCR-positive	Maxilase, indomethacin, phenoxymethylpenicillin, gentamicin
	4-year-old male	Fever, itchy rash, parotitis, dysphagia, lymphadenopathy	NR	Recovered	PCR-positive	Isoprinosine, quinine, amoxicillin, gentamicin, benzylpenicillin, rovamycin
	17-year-old male	Fever, rash, arthralgia, myalgia; patient recently had malaria	NR	Recovered	PCR-negative	Isoprinosine, chloroquine, amoxicillin
	11-year-old male	NR	NR	Recovered	Nil (probable†)	NR
	11-year-old male	NR	NR	Recovered	Orthopoxvirus IgG-positive	NR
	3-year-old male	Rash	Nil	Recovered	Orthopoxvirus IgM and IgG-positive	NR
Sejvar et al (2004)^54^; observational study; USA; n=1						
	6-year-old female	Fever, rash, sore throat, malaise, anorexia, headache, enlarged tonsils, cervical lymphadenopathy	New-onset seizures 6 days after onset of initial illness preceded by somnolence (encephalitis)	NR	PCR-positive (blood), IgM-positive (CSF)	IV antibiotics, lorazepam, phenobarbital
Anderson et al (2003)^55^; case report; USA; n=1						
	“School age” female	Fever, rash, chills, night sweats, fatigue; swollen, painful cervical lymph nodes	Dysphagia, vomiting, difficulty in breathing, inability to eat and drink, retropharyngeal abscess	NR	NR (suspected)	NR
Meyer et al (2002)^7^; outbreak report; DR Congo; n=11						
	1·5-year-old male	Rash on palms, soles, mouth, face, and trunk	NR	NR	PCR-positive (lesion; clade I)	NR
	12-year-old male	Rash on palms, soles, face, and trunk (severe)	NR	Recovered	PCR-positive (clade I)	NR
	3-year-old male	Rash on palms, soles, face, and trunk (severe)	NR	Recovered	PCR-negative	NR
	2-year-old, sex NR	Rash on palms and soles (severe), trunk, and face (mild)	NR	Recovered	PCR-negative	NR
	11-year-old male	Rash on palms and soles (severe), trunk, and face (mild)	NR	Died	PCR-positive (clade I)	NR
	14-year-old male	Rash on palms, sole, face, and trunk; cervical and inguinal lymphadenopathy	NR	NR	PCR-positive (lesion)	NR
	9-year-old female	Fever; rash on palms, soles, face, and trunk; cervical lymphadenopathy	NR	Recovered	PCR-positive (lesion)	NR
	8-year-old male	Fever; rash on palms, soles, face, and trunk; cervical lymphadenopathy	NR	Recovered	PCR-positive (lesion)	NR
	4·5-year-old female	Fever, generalised rash, shivers, cervical lymphadenopathy	NR	Died	NR (suspected)	NR
	3·5-year-old female	Fever, generalised rash, shivers, cervical lymphadenopathy	NR	Died	NR (suspected)	NR
	3·5-year-old male	Generalised rash, conjunctivitis, pharyngitis	Pulmonary failure	Died	NR (suspected)	NR
Tchokoteu et al (1991)^76^; case report; Cameroon; n=1						
	7-year-old male	Fever, rash (face, limbs, scalp, palms, soles, genitalia), abdominal pain, cervical lymphadenopathy, vomiting, headache, facial swelling, stomatitis, dysphagia, anorexia, weakness	Tachypnoea	Recovered	PCR-positive (lesion)	Oral antibiotics, antipyretics, and dietary supplements
Meyer et al (1991)^82^; case report; Gabon; n=5						
	9-month-old female	Fever, skin lesions, mild diarrhoea and vomiting, lymphadenopathy, facial oedema, painful pharyngitis, sleepy, hepatomegaly	Haemorrhagic fever (buccal mucosal bleeding), possibly due to liver failure and coagulopathy	Died	Viral isolation (blood)	Parenteral nutrition, steroids, furosemide
	4-year-old male	Fever, skin lesions, sleepy, clinically unwell, facial oedema, no meningitic signs, lymphadenopathy, hepatomegaly, pharyngitis; had concurrent malaria	Haemorrhagic fever (haematemesis, buccal mucosal bleeding), possibly due to liver failure and coagulopathy	Died	NR (suspected)	Antibiotics, quinine
	9-year-old female	Fever (up to 40°C), sleepy, clinically unwell, facial and neck oedema, skin lesions, lymphadenopathy, splenomegaly, pharyngitis	NR	Recovered	NR (suspected)	Penicillin, aspirin
	6-year-old female	Fever, skin lesions which scar, lymphadenopathy	NR	Recovered	NR (suspected)	Nil
	6-year-old male	NR	NR	NR	NR (suspected)	NR
Herve et al (1989)^61^; outbreak report; Central African Republic; n=2						
	6-year-old female	Rash on trunk, limbs, palms, soles	NR	Recovered	PCR-positive (lesion)	NR
	8-year-old male	Rash on trunk, limbs, palms, soles; severe, large cervical lymphadenopathy; significant weight loss	NR	Recovered	PCR-positive (lesion)	NR
Jezek et al (1986)^42^; case series; Zaire; n=5						
	5-year-old male	Fever, rash (generalised—up to 320 lesions), submandibular lymphadenitis, tonsillitis	Nil	Recovered	Radioimmunoabsorption assay tests positive with convalescent sera	NR
	7-year-old male	Fever, rash (170 lesions, centrifugal, affecting palms and soles)	NR	Recovered	Radioimmunoabsorption assay tests positive with convalescent sera	Antibiotics
	18-month-old male	Fever, generalised rash, oral lesions, submandibular lymphadenopathy (after measles)	Developed respiratory distress syndrome at scabbing stage	Died	Viral isolation (lesion)	NR
	4-year-old female	Fever; centrifugal rash (416 lesions); cervical, auricular, inguinal, and axillary lymphadenopathy; dehydration; weakness (had measles 1 month before)	NR	Recovered	Viral isolation (lesion)	NR
	7-year-old, sex NR	Fever, rash (34 lesions), cervical and inguinal lymphadenopathy (had received smallpox vaccination at birth)	NR	Recovered	Viral isolation (lesion)	NR
Khodakevich et al (1985)^62^; case series; Central African Republic; n=5						
	Age and sex NR; total n=6 (of which n=5 children)	NR; aggregate data: cases had fever, rash (circular deep lesions), lymphadenopathy and mouth lesions in some	NR	NR	EM detected poxvirus particles (lesion)	NR
	Age and sex NR	Deep lesions 4–6 mm yellow-grey in colour, no umbilication; cervical and inguinal lymphadenopathy	NR	NR	EM detected poxvirus particles (lesion)	NR
	Age and sex NR	Deep lesions 4–6 mm yellow-grey in colour, no umbilication; cervical and inguinal lymphadenopathy (painless, 5–8 cm); buccal mucosal lesions 5–6 mm	NR	NR	EM detected poxvirus particles (lesion)	NR
	10-month-old female	Rash, 40% of lesions on back; deep lesions 4–6 mm, yellow-grey in colour, no umbilication; buccal mucosal lesions 5–6 mm	NR	NR	EM detected poxvirus particles (lesion)	NR
	10-year-old female	Rash (generalised including palms), cervical lymphadenopathy	NR	Recovered	EM detected poxvirus particles (lesion)	NR
Janseghers et al (1984)^43^; Zaire; n=1						
	2·5-year-old male	Fever, malaise, rash (vesicles or pustules over arms, legs, palms, soles, genitals, face, mouth, tongue), bilateral conjuctivitis, palpebral lesions, splenomegaly	Developed pneumonia, then high fever and seizures	Died	EM-positive (lesion); viral culture-positive (lesion)	Penicillin, bronchodilators, chloramphenicolsteroids
Mutombo et al (1983)^44^; case report; Zaire; n=1						
	6-month-old female	Fever, generalised rash, inguinal lymphadenopathy (severely bitten by chimpanzee before illness)	NR	Recovered	EM-positive (lesion)	NR
Merouze et al (1983)^77^; case report; Côte d'Ivoire; n=1						
	3-year-old female	Fever; rash on face, scalp, palms, soles; conjunctival secretions	Nil	Recovered	EM-positive (sent to CDC Atlanta) and ELISA	Benzylpenicillin, moroxydine hydrochloride (antiviral)
Breman et al (1977)^78^; outbreak report; Côte d'Ivoire; n=1						
	5-year-old male	Fever; rash on face, all over body including scalp, palms, soles—completely desquamated leaving scars; headache	Nil	Recovered	IFA-positive (late convalescent serum)	NR
Ladnyj et al (1972)^45^; case report; DR Congo; n=1						
	9-month-old female	Fever, rash (haemorrhagic and centrifugal, lasted 2 weeks)	Otitis, mastoiditis, lymphadenitis during crusting stage; the latter required incision and drainage	Just before discharge, developed measles and died	Viral isolation at WHO reference laboratory	NR
Foster et al (1972)^83^; case series identified by surveillance study; Liberia; n=5						
	4-year-old female	Fever, rash (deep, rare coalescence, on palms and soles) sore throat, malaise	NR	Recovered	Viral isolation (lesion)	NR
	4-year-old male	Fever, mild rash (10 lesions)	NR	Recovered	Serology-positive	NR
	6-year-old female	Mild rash (10 lesions)	NR	Recovered	Serology-positive	NR
	9-year-old male	Rash (firm, deep-seated lesions, on face, legs, arms, palms, soles)	Lymphadenitis; pustular eye lesion and scar in cornea	Recovered	Viral isolation (lesion)	Lymphadenitis treated with penicillin
	4-year-old female (only case from Nigeria)	Fever, malaise, headache, sweating, severe prostration, severe generalised rash (firm, discrete, deep lesions, including palms and soles)	Became toxic on 8th day of rash	Recovered	Viral isolation (lesion)	NR
Eke et al (1972)^65^; case report; Nigeria; n=1						
	4-year-old female	Fever, malaise, headache, myalgia, anorexia, prostration, generalised rash (involving palms, soles, and oral mucosa), sore throat, marked splenomegaly	NR	Recovered	Viral culture-positive (lesion)	Penicillin, aspirin, chloroquine, vitamin B complex, iron
**Aggregate data**						
Pittman et al (2023)^46^; observational study; DR Congo; n=170						
	Age range 0–18 years (total n including adults=244)	Aggregate data including adult data: skin lesions (99·5%), lymphadenopathy (98·6%), sore throat (78·2%), anorexia (50·0%), cough (48·1%), and chills (44·5%)	NR	3/170 died	Aggregate including adult data; 216/244 were PCR-positive in both pan-orthopox and mpox-specific PCR	Empirical amoxicillin, antimalarials, mebendazole, analgesia, topical potassium permanganate
Vaughan et al (2022)^87^; surveillance study; Europe‡; n=41						
	Age range 0–17 years	Aggregate data including adult data; 95% had rash and 64·8% also at least one systemic symptom (fever, muscle pain, chills, headache)	2/27 with information were hospitalised	3 cases admitted to ICU, 2 deaths (encephalitis)	NR	NR
Hennessee et al (2022)^56^; surveillance study; USA; n=83						
	16 aged 0–4 years; 12 aged 5–12 years; 55 aged 13–17 years; age 0–12 years, 64% male; age 13–17 years, 89% male	Children aged 0–12 years: lesions mostly occurred on trunk, no anogenital lesions; adolescents: lesions mostly occurred on trunk (33; 60%) and genitals or perianal area (33; 60%)	11% hospitalised, no ICU admissions	No deaths	PCR-positive	In 28 children: 10 (36%) received tecovirimat; 1 (4%) received VIGIV; and 3 (11%) received topical trifluridine; in 55 adolescents: 8 (15%) received tecovirimat
Aguilera-Alonso et al (2022)^68^; surveillance study; Spain; n=16						
	4 aged <4 years, 12 aged 13–17 years; age <4 years: 2 female, 2 male; age 13–17 years: 8 male, 4 female	Rash in 100%, lymphadenopathy in 31%, fever in 25%, asthenia in 13%, sore throat in 13%, myalgia in 6%, vomiting in 6%, diarrhoea in 6%	One child aged <4 years developed bacterial superinfection that required abscess drainage, managed as an outpatient	All recovered	Monkeypox virus or orthopoxvirus generic real-time PCR-positive	NR
Besombes et al (2022)^1^; surveillance study; Central African Republic; n=89						
	63 aged 0–9 years; 26 aged 10–19 years	Aggregate data and including adult data; all (100%) confirmed-case patients had a rash, and most reported fever (93·2%), pruritus (81·5%), and lymphadenopathy (78·6%)	Aggregate and including adult data (keloid scars, septicaemia, bronchopneumonia, dehydration, fistulation of adenopathy)	CFR 8/83 (9·6%) in children aged <16 years (suspected and confirmed cases)	50 confirmed (PCR-positive in blood), 39 suspected (clade I)	NR
Whitehouse et al (2021)^2^; surveillance study; DR Congo; n=707						
	Age range 0–19 years	Aggregate data	Aggregate data	Incidence highest among 5–9-year-olds (18·1 per 100 000)	PCR-positive	Aggregate data
Johnston et al (2015)^47^; observational study; DR Congo; n=14						
	Age range 1·5–17 years	Mild is <25 lesions, moderate is 25–99, severe is 100–250, serious is >250; 4 patients had serious disease, 4 severe, 4 moderate, and 2 mild	MIP-1 alpha and beta are elevated in cases of mild disease compared with moderate and severe; GM-CSF, IL-10, sIL-2R in extremely high concentrations in samples from serious disease	NR	Observational study	NR
Hutin et al (2001)^5^; outbreak report; DR Congo; n=88						
	Aggregate	NR	NR	3/81 cases with follow-up died—all in children <3 years old	Aggregate: all 7 with active lesions were PCR-positive; 89% of all cases were orthopoxvirus antibody-positive	NR
CDC (1997)^51^; outbreak report; Zaire; total n=92 (n=3 died)						
	Aggregate; age range 0–3 years	Rash (that did not look like chickenpox in an area with a chickenpox outbreak)	NR	3 out of 3 that died were children	NR (suspected)	NR
Jezek et al (1988)^49^; surveillance study; Zaire; n=315						
	Age range 0–14 years	Aggregate data§	Aggregate data but included blindness and “weak vision”	33 deaths were all in children aged 3 months to 8 years; CFR=14·5% (0–4 year-olds) and 7·5% (5–9 year-olds)	Cases had “laboratory testing” of skin lesions or serum (probable)	NR
Jezek et al (1988)^48^; surveillance study; Zaire; n=85						
	Age range 0–14 years	Aggregate data; study period: 1981–85 in Bumba region, Zaire—possible overlap with above study	NR	8 deaths (6 girls and 2 boys aged 7 months to 8 years)	Virus detection in 68 (lesion); serology in 20 and 3 died before specimen collection	NR
Jezek et al (1987)^23^; surveillance study; Zaire; n=263						
	Aggregate data 263 children (total n=282)	Aggregate data	Aggregate data but included: encephalitis, septicaemia, broncho-pneumonia, vomiting, diarrhoea and dehydration, keratitis, corneal opacities leading to impaired vision (a 4-year-old ended up with bilateral blindness and an 11 month-old and two 5-year-olds with unilateral blindness), scars deforming eyelids and nares, keloids, and alopecia	One child (aged 3 years) died of encephalitis and a 5-year-old with >4500 lesions died of septicaemia; 19 children died of bronchopneumonia	NR	NR
Jezek et al (1987)^50^; serological survey; Zaire; n=19 seropositive						
	Aggregate data; total n=3460; n=27 total seropositive; n=19 seropositive children	Aggregate data; 7/19 seropositive children had no scars or history of disease	NR	NR	Serology	NR
Breman et al (1980)^88^; outbreak report; west and central Africa; n=40; total n=47						
	Aggregate data; children n=40 aged 7 months to 7 years	Aggregate data	NR	All 8 that died were children aged 7 months to 7 years	Virological or serological tests were done in all	NR

“n” denotes the number of paediatric cases with data available in each study. Of the countries included in this table, mpox is non-endemic in Brazil, France, the Netherlands, Spain, the UK, and the USA, and endemic in Cameroon, Central African Republic, Côte d'Ivoire, DR Congo (formerly Zaire), Gabon, Liberia, Nigeria, Republic of the Congo, Sierra Leone, and Sudan. CDC=Centers for Disease Control and Prevention. CFR=case fatality ratio. CSF=cerebrospinal fluid. EM=electron microscopy. ICU=intensive care unit. IFA=immunofluorescence assay. IgG=immunoglobulin G. IgM=immunoglobulin M. IV=intravenous. NR=not reported. VIGIV=vaccinia immune globulin intravenous.

* Tecovirimat was considered but discounted as there is no licence or dosing for patients <13 kg.

† Probable case=one epidemiological factor plus fever and typical rash appearing less than 21 days after contact with an unwell animal or confirmed, probable, or suspected case.

‡ Reporting countries: Andorra, Austria, Belgium, Bosnia and Herzegovina, Bulgaria, Croatia, Cyprus, Czechia, Denmark, Estonia, Finland, France, Georgia, Germany, Greece, Hungary, Iceland, Ireland, Israel, Italy, Latvia, Lithuania, Luxembourg, Malta, the Netherlands, Norway, Poland, Portugal, Moldova, Romania, Slovakia, Slovenia, Spain, Sweden, Switzerland.

§ Significantly higher proportions of confluent exanthem, more numerous skin lesions, and more frequent enanthem in the oral cavity were found in patients infected from an animal source. However, there were no significant differences between those infected by an animal source, and those infected from human-to-human transmission in the duration of illness, frequency of complications and sequelae, or severity of illness, as characterised by the extent of the body lesions, intensity of systemic symptoms, physical incapacity and need for special care, or the crude CFRs.

**Table 2:** Details of all included maternal and breastfeeding studies

		**Clinical features and complications**	**Outcome**	**Laboratory diagnosis**	**Treatment**
**Maternal studies**					
Sampson et al (2023)^57^; case report; USA; n=1					
	Age of pregnant person: 20 years; gestational age when infected: 31 weeks	Labial ulcer and papular rash	Live birth at 39 weeks and 2 days (completely asymptomatic)	Neonate IgG-positive	Tecovirimat given to mother
Oakley et al (2023)^11^; observational study; USA; n=23					
	Age of pregnant person: 20–35 years; gestational age when infected: among 10 cases with known trimester of infection: 3/10 (30%) occurred during the first, 4/10 (40%) during the second, and 3/10 (30%) during the third trimester	4/10 (17·4%) were hospitalised (pain control and treatment of cellulitis) and remained pregnant at discharge; none required intensive care, intubation, or unplanned delivery	3/21 had reported outcomes; 2/3 had full-term deliveries (no complications, no mother-to-child transmission); 1/3 miscarriage at 11 weeks	NR	11/23 (48%) received tecovirimat (administered during all trimesters of pregnancy); no medication-related adverse events
Ogoina et al (2020)^21^; retrospective observational study; Nigeria; n=1					
	Age of pregnant person: NR; gestational age when infected: 16 weeks (note: unknown if HIV-positive as 33·3% of women [3/9] were in this study)	NR (aggregate)	PROM at 16 weeks and intrauterine fetal death	Nil (suspected case)	NR
Yinka-Ogunleye et al (2019)^64^; surveillance study; Nigeria; n=1					
	Age of pregnant person: NR; gestational age when infected: 26 weeks	Vesiculopustular rash	Spontaneous miscarriage at 26 weeks	PCR-positive or IgM-positive (aggregate)	NR
Mbala et al (2017)^8^; observational study; DR Congo; n=4					
	Age of pregnant person: 20 years; gestational age when infected: 6 weeks	Vesiculopustular rash (76 lesions)	Miscarriage at 6 weeks	Maternal PCR-positive	Antibiotics, mebendazole, quinine
	Age of pregnant person: 25 years; gestational age when infected: 6–7 weeks	Vesiculopustular rash (1335 lesions)	Miscarriage at 6–7 weeks	Maternal PCR-positive	Antibiotics, mebendazole, quinine
	Age of pregnant person: 29 years; gestational age when infected: 14 weeks	Vesiculopustular rash (16 lesions)	Live birth	Maternal PCR-positive	Antibiotics, mebendazole, quinine
	Age of pregnant person: 22 years; gestational age when infected: 18 weeks	Vesiculopustular rash (113 lesions)	Fetal death	Maternal, placental, and fetal tissue PCR-positive	Antibiotics, mebendazole, quinine
Doshi et al (2019)^72^; outbreak report; Republic of the Congo; n=1					
	Age of pregnant person: 33 years; gestational age when infected: “term”	NR	Admitted to hospital: outcome: “alive”	Orthopoxvirus IgG and IgM positive	NR
Jezek et al (1983)^9^; case report; DR Congo; n=1					
	Age of pregnant person: NR; gestational age when infected: NR	Baby had neonatal rash, consistent with mpox	Died at 6·5 weeks of age (malnutrition)	NR	NR
**Breastfeeding studies**					
Oakley et al (2023)^11^; observational study; USA; n=3					
	Age of child: 4 days	Maternal lesions including under breasts; newborn developed lesions on chest and face 6 days later; complications NR	Infant infected	NR	NR
	Age of child: NR	Developed “atypical” features of mpox after caring for mpox patient (health-care worker); breastfeeding at the time of mpox diagnosis; complications NR	NR	Breast milk PCR-negative	NR
Alonso-Cadenas et al (2023)^13^; case report; Spain; n=1					
	Age of child: 7 months	Fever, rash (10 lesions), hyperaemic pharynx and petechial enanthema; complications NR	Survived	PCR-positive in lesion (breast milk not tested)	Nil
Nakoune et al (2017)^12^; outbreak report; Central African Republic; n=1					
	Age of child: 15 months	Rash; was breastfeeding from mother but mother and siblings already unwell; complications NR	Died	NR	NR

Of the countries included in this table, mpox is non-endemic in Spain and the USA, and endemic in Central African Republic, DR Congo, and Republic of the Congo. IgG=immunoglobulin G. IgM=immunoglobulin M. NR=not reported. PROM=premature rupture of membranes.

39 studies reported individual paediatric outcomes in a total of 101 cases, of which 89 (88%) were from endemic countries (ie, countries where mpox cases were reported before the 2022 outbreak). 14 additional studies reported aggregated data for paediatric outcomes of interest among a total of 2022 cases, of which 1882 (93%) were from endemic countries, with potential overlap of cases in three studies (n=263,^23^ n=85,^48^ n=315^49^) carried out in overlapping time periods (1980–86) and regions (formerly Zaire, present day DR Congo).

Age was reported for 97 of 101 individual cases and ranged from 9 days to 18 years (median 4·5 years [IQR 2–9 years]; appendix 4 p 5). The median age of cases from non-endemic countries was lower compared with endemic countries (18 months [IQR 2 months to 8 years] *vs* 5 years [IQR 3–9 years]).

Among 101 paediatric cases, 59 (58%) were confirmed; of these 59, 42 (71%) were diagnosed by PCR (lesion or throat swab or blood) and 17 (29%) by viral isolation or culture of lesion fluid. An additional 16 (16%) of 101 cases met the probable case criteria and had positive immunoglobulin M (IgM) or immunoglobulin G (IgG) OPXV serology. The remaining 23 (23%) of 101 cases were probable or suspected cases—ie, clinical cases with or without positive OPXV serology (see appendix 4 pp 2–3 for full definitions).

Viral clade was available for six (7%) of 89 endemic cases (two clade II, four clade I) and for four (33%) of 12 non-endemic cases (two clade II, two clade IIb).

**Figure 3:**
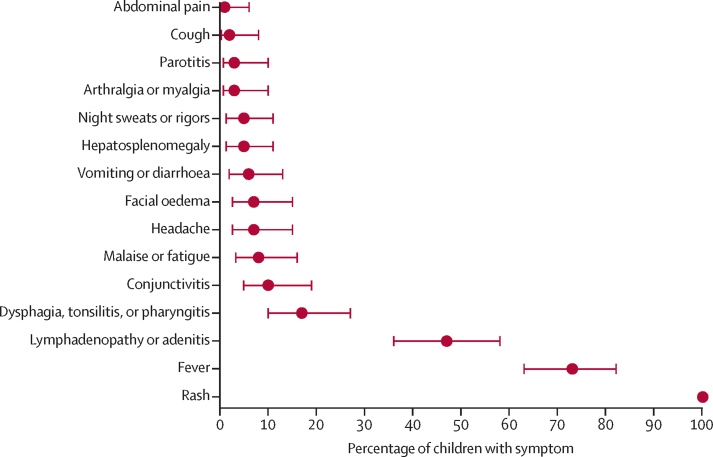
Reported signs and symptoms of paediatric mpox cases (n=86) Error bars show 95% CIs.

Symptom data were available in 86 of 101 children, in whom the most common symptoms were skin lesions (86 [100%]); fever (63 [73%; 95% CI 63–82]); lymphadenopathy or adenitis (40 [47%; 36–58]); dysphagia, tonsillitis, or pharyngitis (15 [17%; 10–27]); and conjunctivitis (nine [10%; 5–19]; figure 3). Among non-endemic cases, fever and lymphadenopathy were less frequent than among endemic cases (fever: one of 12 [8%; 95% CI 0–38] *vs* 62 of 74 [84%; 73–91]; lymphadenopathy: two of 12 [17%; 2–48] *vs* 38 of 74 [51%; 39–63]), and genital rash was present in one 18-year-old patient in France (appendix 4 p 6).

Complications were reported in 20 of 101 paediatric cases (20% [95% CI 13–29]). Among these 20 cases, bacterial infective complications occurred in 12 (60% [36–81]; table 1 and appendix 4 p 6). Ophthalmic complications such as corneal scarring were reported in two (10% [1–32]).^52^, ^83^

Large surveillance studies from DR Congo (n=263)^23^, ^49^ also reported on ophthalmic complications, with one reporting bilateral visual loss in one child and unilateral blindness in three children secondary to keratitis and corneal opacities.^23^

Tecovirimat use was reported in 21 patients aged 9 days to 17 years,^52^, ^53^, ^56^, ^69^ from the USA (n=20) and UK (n=1), with two possible overlapping cases.^52^, ^56^ Complications prompted treatment in some cases, including respiratory disease (respiratory failure requiring ventilation with secondary adenoviral infection; intravenous cidofovir was also given),^69^ ocular disease (corneal lesions and periorbital cellulitis),^52^ and cellulitis with eyelid lesions (VIGIV was also given).^53^ Other treatments used were supportive (table 1 and appendix 4 p 6).

Outcome data were available for 84 of 101 children, of whom 20 (24% [95% CI 15–34]) died. Sequential infections and co-infections featured in several cases. Two siblings died of coagulopathy and haemorrhage, one of whom had concurrent malaria in a study in Gabon,^82^ and one child hospitalised for malaria developed mpox in hospital (presumed nosocomial infection in Republic of the Congo).^74^ One child with mpox developed measles shortly before discharge and died in hospital;^45^ two other children who died^42^, ^43^ had measles shortly before becoming unwell with mpox in Republic of the Congo and DR Congo, respectively. One adolescent in the USA was diagnosed with concurrent HIV.^56^

A larger study looking at causes of death in 21 children with mpox in DR Congo^23^ reported that 19 children died of bronchopneumonia, one of encephalitis, and one of septicaemia.

**Figure 4:**
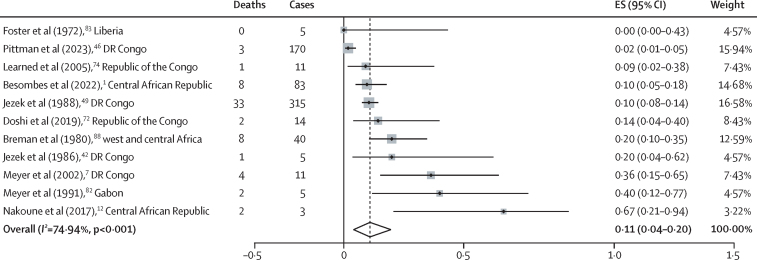
Forest plot of paediatric mpox CFRs reported by 11 studies CFR=case fatality ratio. ES=effect size (in this case CFR).

Paediatric mortality data were pooled from seven outbreak reports or small case series^7^, ^12^, ^42^, ^72^, ^74^, ^82^, ^83^ and four surveillance studies or large case series, all from endemic countries.^1^, ^46^, ^49^, ^88^ CFRs ranged from 0% to 67%, with a pooled estimate of 11% (95% CI 4–20), *I*^2^=75% (figure 4).

Seven studies reported mpox in 32 pregnant individuals (table 2) from the USA (n=24, 75% of cases), DR Congo (n=5), Republic of the Congo (n=1), and Nigeria (n=2). Infections occurred between 6 weeks' and 31 weeks' gestation. In two of these 32 individuals, symptoms developed 3 days after delivery in a US study^11^ (included as maternal cases given the mpox incubation period of 5–21 days).^89^

Laboratory confirmation was available for 30 (94%) of 32 pregnancies. Among the 32 pregnancies, PCR positivity was confirmed in four (13%), and 24 (75%) were either PCR-positive or met the “probable” case laboratory criteria.^90^ Viral clade was not available for any maternal cases. Two cases (6%) had only serological test results.^57^ The remaining two (6%) had a clinical diagnosis.

An observational study of 23 pregnant cisgender women with mpox in the USA reported rash in 23 (100%; 17% with genital or breast lesions), fever in six (26%), lymphadenopathy in three (13%), and myalgia in two (9%).^11^ Individual symptom data from case reports for six maternal cases (table 2)^8^, ^57^, ^64^ only described a vesiculopustular rash (one had genital lesions).^57^ In one case in DR Congo, maternal symptoms were absent, but at birth the neonate was reported to have an mpox rash.^9^

Tecovirimat was used in 12 pregnant individuals throughout all trimesters in the USA.^11^, ^57^ No medication-related adverse events were reported. Two maternal cases in the USA occurred 3 days after delivery and their newborns developed lesions up to 1 week later (these might represent congenital or postnatal infections).^11^ Both newborns received oral tecovirimat for 10–14 days (one also received VIGIV), responded to treatment, and were discharged. Four pregnant individuals in DR Congo were given supportive treatments including antimicrobials.^8^

Outcome data were available for 12 of 32 pregnancies, of which six (50%; 95% CI 21–79), resulted in fetal death between 6 weeks' and 26 weeks' gestation in studies from DR Congo (n=3),^8^ Nigeria (n=2),^21^, ^64^ and the USA (n=1).^11^ The remaining six patients had live births at term. One infant in DR Congo died of malnutrition at 6·5 weeks of age.^9^ There were no maternal deaths. It was not possible to conduct a pooled estimate of fetal CFR as most studies were single case reports.

Post-mortem and pathology findings were available for one stillborn fetus delivered at 18 weeks' gestation by a patient in DR Congo with notable mpox viraemia (10^6^ copies per mL; equivalent to cycle threshold of 22).^8^, ^91^, ^92^ The fetus had diffuse cutaneous maculopapillary lesions on the head, trunk, and extremities. Hydrops fetalis was detected, with marked hepatomegaly and a peritoneal effusion. The placenta had haemorrhages. This mother was also found to have malaria.^8^

Three case studies reported outcomes of four breastfeeding infants whose mothers had mpox in the USA (n=3),^11^ Central African Republic (n=1),^12^ and Spain (n=1; table 2).^13^ Breastmilk underwent PCR testing in one case^11^ and was negative for MPXV (the outcome for this case was not reported).^11^ The three infants with known outcomes were all infected and developed a rash^11^, ^12^, ^13^ and one died.^12^ Lesions on the chest were specifically reported in three cases.^11^, ^12^, ^13^ No specific antiviral treatments were given.

Two studies reported on the use of one dose of live, non-replicating vaccine to treat a total of 28 children (appendix 4 p 7).^58^, ^71^ The MVA-BN vaccine (IMVANEX) was administered to 21 children across three school and nursery outbreaks in the UK in 2022: seven aged 2–3 years, four aged 4–5 years, and ten aged 5–11 years.^71^ MVA-BN (JYNNEOS) vaccine and VIGIV were administered to seven children and one neonate, respectively, in two outbreaks in the USA, as post-exposure prophylaxis.^58^ There were no reported side-effects and no children developed mpox.

No studies published the experience of third-generation vaccine use during pregnancy or breastfeeding.

## Discussion

Our data present the largest collection of paediatric and maternal mpox cases to date. Estimates of pooled paediatric CFR point to substantial mortality in children of up to 11% (95% CI 4–20) in endemic countries. Maternal infection results in fetal loss in 50% (95% CI 21–79) of cases (six of 12) across all trimesters. The use of antiviral and immunological treatments including tecovirimat, cidofovir, and VIGIV in childhood and pregnancy is supported by a small number of case reports. Finally, the third-generation vaccine was used uneventfully in a small paediatric population in the UK and USA.

Our data on the clinical features of paediatric mpox cases, the majority of which were from endemic countries (88%), are consistent with larger studies in endemic countries, indicating that in children, fever for 1–3 days before rash appearance and lymphadenopathy are common.^1^ This contrasts with clinical features reported in adolescents and young adults in the 2022 outbreak, where systemic features are rare and genital lesions common.^93^ These differences, which were also perceived among non-endemic cases in our study, are probably due to the viral clade (primarily IIb) and mode of transmission (through intimate contact) observed in the 2022 outbreak.^93^

Our pooled paediatric CFR obtained from studies in endemic countries showed high interstudy heterogeneity. Mpox CFR is known to vary by age (15% in 0–4-year-olds *vs* 8% in 5–9-year-olds)^49^ and viral clade. Clade I has a higher CFR (10·6%) than clade IIa and IIb (3·6%),^94^, ^95^ factors we were unable to control for in our review due to small numbers and unavailability of data.

Fetal death occurred in half of the pregnancies studied in this review, lower than reported in a previous study where it was noted to be as high as 77%.^20^ Additional data are awaited, including from large Brazilian datasets, to study this further.^96^

An understudied and probable contributary factor to the poor outcomes of paediatric and maternal mpox is the impact of concurrent or sequential infections.^3^ Among endemic cases, malaria was implicated in one maternal case resulting in a second trimester miscarriage,^8^ and five of 20 children who died had been recently diagnosed with measles^42^, ^43^, ^45^ or malaria.^74^, ^82^ HIV status was infrequently reported among paediatric cases,^56^ but is known to cause prolonged illness, larger lesions, and higher frequency of genital ulcers among adults and should be further studied in children.^21^

There are no completed randomised controlled trials investigating treatments for mpox, although five trials are ongoing, testing the effectiveness of tecovirimat, some of which include children.^97^ Low-level evidence has indicated a possible safety signal from brincidofovir resulting in mild liver injury in three individuals.^97^ VIGIV has been shown to be safe in immunosuppressed mice^98^, ^99^ and has been used to treat severe mpox in people living with HIV unresponsive to tecovirimat.^100^ Our data, from the USA and UK, on the experience of tecovirimat, cidofovir, and VIGIV use in children aged 0–17 years,^53^, ^69^ and tecovirimat use in pregnant individuals, provides some initial evidence of their utility.

Our study also found limited evidence, from the USA and UK, that live non-replicating smallpox vaccines can be given uneventfully for the prevention of mpox in children.^58^, ^71^ Recent data from seven children given the MVA-BN vaccine in the UK have also shown adequate antibody and cellular immune responses up to 15 weeks after vaccination.^101^

The key strengths of this study include the robust methodological approach and the meta-analysis of available data to provide the first contemporary estimates for mpox CFR in children. The evidence base does, however, have limitations. No randomised controlled trials or cohort studies were available, only observational studies (some with very low case numbers) and case reports. There was a reliance on clinical criteria for the detection of some cases and this might have affected the accurate calculation of incidence of infection and complications. The limitations in the ascertainment of the denominator might have also affected our meta-analysis and led to inflation of the CFR.^102^ Other potential limitations are the variability in the size of included studies, resulting in uneven weight contribution to the pooled estimate, and the inclusion of studies with small (n≤5) case numbers and the risk of “small study effects”.^103^

The ability to collect high-quality data in endemic regions is hampered by armed conflict and flooding^104^ affecting the highly endemic areas of Kasaï Oriental, North Kivu, and South Kivu in eastern DR Congo for over 30 years.^105^, ^106^ This has resulted in large-scale population displacement. As mobile populations move into forested areas, challenges in surveillance and control are compounded by a greater risk of zoonotic mpox transmission. Coupled with an increase in susceptible individuals since the cessation of the smallpox vaccination programme, this is likely to result in a further increase in mpox incidence.^3^, ^4^, ^107^, ^108^, ^109^, ^110^

Several recommendations are proposed. Firstly, funding and priority should be given to mpox surveillance (including improved zoonotic surveillance) and the collection of disaggregated longitudinal data in endemic regions. The creation of a global registry of cases is therefore indicated, to facilitate the further study of at-risk groups such as children and pregnant women and the role of co-factors that could worsen outcomes such as co-infections, genetic variants, and social determinants of health. Secondly, in parallel, scaling up of free testing with government backing must occur to support adequate case detection.^111^ Lastly, endemic populations must be included in clinical trials assessing novel treatments and vaccines. This includes appropriate vaccine allocation and distribution, prioritising the most at-risk groups in the endemic countries. These recommendations are in line with the WHO mpox strategic objectives to: (1) interrupt human-to-human transmission, with a focus on high-risk populations; (2) minimise zoonotic transmission; and (3) protect vulnerable groups at risk of severe mpox disease.^112^

These tasks will require close collaboration with stakeholders in endemic countries in global partnerships and the leveraging and re-purposing of existing technologies (eg, contact tracing or surveillance applications, mobile vaccine passports, and rapid PCR diagnostics) developed during the COVID-19 pandemic. We urge the international community to work collaboratively and think equitably about how we focus our research efforts as the mpox pandemic continues, to benefit the most at-risk populations in endemic countries.

## Data sharing

Data collected for the study including the data collection tool, the study protocol, search terms, and any code used for the analysis will be shared with individuals upon reasonable request to the corresponding author from the time of publication of the Article.

## Declaration of interests

SBD has received honoraria from MSD and Sanofi for taking part in respiratory syncytial virus (RSV) advisory boards and has provided consultancy and/or investigator roles in relation to product development for Janssen, AstraZeneca, Pfizer, Moderna, Valneva, MSD, iLiAD, and Sanofi with fees paid to St George's, University of London. SBD is a member of the UK Department of Health and Social Care's (DHSC) Joint Committee on Vaccination and Immunisation (JCVI) RSV subcommittee and Medicines and Healthcare products Regulatory Agency's (MHRA) Paediatric Medicine Expert Advisory Group (PMEAG), but the reviews expressed herein do not necessarily represent those of DHSC, JCVI, MHRA, or PMEAG. All other authors declare no competing interests.

## Supplementary Materials

French translation of the abstract

Spanish translation of the abstract

Portuguese translation of the abstract

## References

[1] Besombes C, Mbrenga F, Schaeffer L (2022). National monkeypox surveillance, Central African Republic, 2001–2021. Emerg Infect Dis.

[2] Whitehouse ER, Bonwitt J, Hughes CM (2021). Clinical and epidemiological findings from enhanced monkeypox surveillance in Tshuapa Province, Democratic Republic of the Congo during 2011–2015. J Infect Dis.

[3] Beer EM, Rao VB (2019). A systematic review of the epidemiology of human monkeypox outbreaks and implications for outbreak strategy. PLoS Negl Trop Dis.

[4] Rimoin AW, Mulembakani PM, Johnston SC (2010). Major increase in human monkeypox incidence 30 years after smallpox vaccination campaigns cease in the Democratic Republic of Congo. Proc Natl Acad Sci USA.

[5] Hutin YJ, Williams RJ, Malfait P (2001). Outbreak of human monkeypox, Democratic Republic of Congo, 1996 to 1997. Emerg Infect Dis.

[6] Khodakevich L, Jezek Z, Messinger D (1988). Monkeypox virus: ecology and public health significance. Bull World Health Organ.

[7] Meyer H, Perrichot M, Stemmler M (2002). Outbreaks of disease suspected of being due to human monkeypox virus infection in the Democratic Republic of Congo in 2001. J Clin Microbiol.

[8] Mbala PK, Huggins JW, Riu-Rovira T (2017). Maternal and fetal outcomes among pregnant women with human monkeypox infection in the Democratic Republic of Congo. J Infect Dis.

[9] Jezek Z, Gromyko AI, Szczeniowski MV (1983). Human monkeypox. J Hyg Epidemiol Microbiol Immunol.

[10] Schwartz DA, Ha S, Dashraath P, Baud D, Pittman PR, Adams Waldorf KM (2023). Mpox virus in pregnancy, the placenta, and newborn. Arch Pathol Lab Med.

[11] Oakley LP, Hufstetler K, O'Shea J (2023). Mpox cases among cisgender women and pregnant persons—United States, May 11– November 7, 2022. MMWR Morb Mortal Wkly Rep.

[12] Nakoune E, Lampaert E, Ndjapou SG (2017). A nosocomial outbreak of human monkeypox in the Central African Republic. Open Forum Infect.

[13] Alonso-Cadenas JA, Andina-Martínez D, García-García CJ, Gaitero-Tristán J, García-Ascaso MT, Torrelo A (2023). Monkeypox disease in a breastfeeding infant. Pediatr Dermatol.

[14] Iñigo Martínez J, Gil Montalbán E, Jiménez Bueno S (2022). Monkeypox outbreak predominantly affecting men who have sex with men, Madrid, Spain, 26 April to 16 June 2022. Euro Surveill.

[15] Thornhill JP, Barkati S, Walmsley S (2022). Monkeypox virus infection in humans across 16 countries—April–June 2022. N Engl J Med.

[16] Adler H, Gould S, Hine P (2022). Clinical features and management of human monkeypox: a retrospective observational study in the UK. Lancet Infect Dis.

[17] Low N, Bachmann LH, Ogoina D (2023). Mpox virus and transmission through sexual contact: defining the research agenda. PLoS Med.

[18] WHO (2022). WHO Director-General declares the ongoing monkeypox outbreak a Public Health Emergency of International Concern. https://www.who.int/europe/news/item/23-07-2022-who-director-general-declares-the-ongoing-monkeypox-outbreak-a-public-health-event-of-international-concern.

[19] WHO (2022). Fifth meeting of the International Health Regulations (2005) (IHR) Emergency Committee on the Multi-Country Outbreak of mpox (monkeypox). https://www.who.int/news/item/11-05-2023-fifth-meeting-of-the-international-health-regulations-(2005)-(ihr)-emergency-committee-on-the-multi-country-outbreak-of-monkeypox-(mpox).

[20] D'Antonio F, Pagani G, Buca D, Khalil A (2023). Monkeypox infection in pregnancy: a systematic review and metaanalysis. Am J Obstet Gynecol MFM.

[21] Ogoina D, Iroezindu M, James HI (2020). Clinical course and outcome of human monkeypox in Nigeria. Clin Infect Dis.

[22] Sejvar JJ, Labutta RJ, Chapman LE, Grabenstein JD, Iskander J, Lane JM (2005). Neurologic adverse events associated with smallpox vaccination in the United States, 2002–2004. JAMA.

[23] Jezek Z, Szczeniowski M, Paluku KM, Mutombo M (1987). Human monkeypox: clinical features of 282 patients. J Infect Dis.

[24] Gruber MF (2022). Current status of monkeypox vaccines. NPJ Vaccines.

[25] UK Health Security Agency (2013).

[26] Beeson AM, Haston J, McCormick DW (2023). Mpox in children and adolescents: epidemiology, clinical features, diagnosis, and management. Pediatrics.

[27] Rao AK, Petersen BW, Whitehill F (2022). Use of JYNNEOS (smallpox and monkeypox vaccine, live, nonreplicating) for preexposure vaccination of persons at risk for occupational exposure to orthopoxviruses: recommendations of the Advisory Committee on Immunization Practices—United States, 2022. MMWR Morb Mortal Wkly Rep.

[28] Japanese Registry of Clinical Trials (2022). A single-arm study to evaluate the immunogenicity and safety of smallpox vaccine as vaccination to monkeypox in Japanese healthy adults. https://jrct.niph.go.jp/en-latest-detail/jRCTs031220171.

[29] Titanji BK, Eick-Cost A, Partan ES (2023). Effectiveness of smallpox vaccination to prevent mpox in military personnel. N Engl J Med.

[30] Lederman E, Miramontes R, Openshaw J (2009). Eczema vaccinatum resulting from the transmission of vaccinia virus from a smallpox vaccinee: an investigation of potential fomites in the home environment. Vaccine.

[31] Kamboj M, Sepkowitz KA (2007). Risk of transmission associated with live attenuated vaccines given to healthy persons caring for or residing with an immunocompromised patient. Infect Control Hosp Epidemiol.

[32] Su JR, McNeil MM, Welsh KJ (2021). Myopericarditis after vaccination, Vaccine Adverse Event Reporting System (VAERS), 1990–2018. Vaccine.

[33] Lesser D (2004). Risk of contact vaccinia from immunization sites. JAMA.

[34] Badell ML, Meaney-Delman D, Tuuli MG (2015). Risks associated with smallpox vaccination in pregnancy: a systematic review and meta-analysis. Obstet Gynecol.

[35] Hoy SM (2018). Tecovirimat: first global approval. Drugs.

[36] Russo AT, Grosenbach DW, Chinsangaram J (2021). An overview of tecovirimat for smallpox treatment and expanded anti-orthopoxvirus applications. Expert Rev Anti Infect Ther.

[37] Moher D, Liberati A, Tetzlaff J, Altman DG (2009). Preferred reporting items for systematic reviews and meta-analyses: the PRISMA statement. PLoS Med.

[38] WHO (2022).

[39] StataCorp (2021).

[40] Murad MH, Sultan S, Haffar S, Bazerbachi F (2018). Methodological quality and synthesis of case series and case reports. BMJ Evid Based Med.

[41] Eltvedt AK, Christiansen M, Poulsen A (2020). A case report of monkeypox in a 4-year-old boy from the DR Congo: challenges of diagnosis and management. Case Rep Pediatr.

[42] Jezek Z, Arita I, Mutombo M, Dunn C, Nakano JH, Szczeniowski M (1986). Four generations of probable person-to-person transmission of human monkeypox. Am J Epidemiol.

[43] Janseghers L, Matamba M, Colaert J, Vandepitte J, Desmyter J (1984). Fatal monkeypox in a child in Kikwit, Zaire. Ann Soc Belg Med Trop.

[44] Mutombo M, Arita I, Jezek Z (1983). Human monkeypox transmitted by a chimpanzee in a tropical rain-forest area of Zaire. Lancet.

[45] Ladnyj ID, Ziegler P, Kima E (1972). A human infection caused by monkeypox virus in Basankusu Territory, Democratic Republic of the Congo. Bull World Health Organ.

[46] Pittman PR, Martin JW, Kingebeni PM (2023). Clinical characterization and placental pathology of mpox infection in hospitalized patients in the Democratic Republic of the Congo. PLoS Negl Trop Dis.

[47] Johnston SC, Johnson JC, Stonier SW (2015). Cytokine modulation correlates with severity of monkeypox disease in humans. J Clin Virol.

[48] Jezek Z, Grab B, Paluku KM, Szczeniowski MV (1988). Human monkeypox: disease pattern, incidence and attack rates in a rural area of northern Zaire. Trop Geogr Med.

[49] Jezek Z, Grab B, Szczeniowski M, Paluku KM, Mutombo M (1988). Clinico-epidemiological features of monkeypox patients with an animal or human source of infection. Bull World Health Organ.

[50] Jezek Z, Nakano JH, Arita I, Mutombo M, Szczeniowski M, Dunn C (1987). Serological survey for human monkeypox infections in a selected population in Zaire. J Trop Med Hyg.

[51] Centers for Disease Control and Prevention (1997). Human monkeypox—Kasai Oriental, Zaire, 1996–1997. MMWR Morb Mortal Wkly Rep.

[52] Ditta LC, Wojcik P, Minniear TD (2023). Ocular presentation of Mpox in a healthy child without known exposure. J AAPOS.

[53] Saunders KE, Van Horn AN, Medlin HK (2022). Monkeypox in a young infant—Florida, 2022. MMWR Morb Mortal Wkly Rep.

[54] Sejvar JJ, Chowdary Y, Schomogyi M (2004). Human monkeypox infection: a family cluster in the midwestern United States. J Infect Dis.

[55] Anderson MGJM, Frenkel LDM, Homann S, Guffey J (2003). A case of severe monkeypox virus disease in an American child: emerging infections and changing professional values. Pediatr Infect Dis J.

[56] Hennessee I, Shelus V, McArdle CE (2022). Epidemiologic and clinical features of children and adolescents aged <18 years with monkeypox—United States, May 17–September 24, 2022. MMWR Morb Mortal Wkly Rep.

[57] Sampson MM, Magee G, Schrader EA (2023). Mpox (monkeypox) infection during pregnancy. Obstet Gynecol.

[58] Minhaj FS, Petras JK, Brown JA (2022). Orthopoxvirus testing challenges for persons in populations at low risk or without known epidemiologic link to monkeypox—United States, 2022. MMWR Morb Mortal Wkly Rep.

[59] Besombes C, Gonofio E, Konamna X (2019). Intrafamily transmission of monkeypox virus, Central African Republic, 2018. Emerg Infect Dis.

[60] Kalthan E, Tenguere J, Ndjapou SG (2018). Investigation of an outbreak of monkeypox in an area occupied by armed groups, Central African Republic. Med Mal Infect.

[61] Herve VMA, Belec L, Yayah G, Georges AJ (1989). [Monkeypox in central Africa. About two strains isolated in Central African Republic]. Med Mal Infect.

[62] Khodakevich L, Widy-Wirski R, Arita I, Marennikova SS, Nakano J, Meunier D (1985). Monkeypox in the Central African Republic. Bull Soc Pathol Exot.

[63] Ogoina D, Izibewule JH, Ogunleye A (2019). The 2017 human monkeypox outbreak in Nigeria—report of outbreak experience and response in the Niger Delta University Teaching Hospital, Bayelsa State, Nigeria. PLoS One.

[64] Yinka-Ogunleye A, Aruna O, Dalhat M (2019). Outbreak of human monkeypox in Nigeria in 2017–18: a clinical and epidemiological report. Lancet Infect Dis.

[65] Eke RA (1972). Monkey-pox in a four-year old girl: case report. West Afr Med J Niger Pract.

[66] Roguera Sopena M, Naqui Xicota L, Hernández Rodríguez Á, Méndez Hernández MJ, Rodrigo Gonzalo de Liria C (2022). Monkeypox, also in pediatric age. An Pediatr (Engl Ed).

[67] Fuente SM, Nava FB, Valerio M, Veintimilla C, Aguilera-Alonso D (2022). A call for attention: pediatric monkeypox case in a context of changing epidemiology. Pediatr Infect Dis J.

[68] Aguilera-Alonso D, Alonso-Cadenas JA, Roguera-Sopena M, Lorusso N, Miguel LGS, Calvo C (2022). Monkeypox virus infections in children in Spain during the first months of the 2022 outbreak. Lancet Child Adolesc Health.

[69] Ramnarayan P, Mitting R, Whittaker E (2022). Neonatal monkeypox virus infection. N Engl J Med.

[70] Hobson G, Adamson J, Adler H (2021). Family cluster of three cases of monkeypox imported from Nigeria to the United Kingdom, May 2021. Euro Surveill.

[71] Ladhani SN, Aiano F, Edwards DS (2022). Very low risk of monkeypox among staff and students after exposure to a confirmed case in educational settings, England, May to July 2022. Euro Surveill.

[72] Doshi RH, Guagliardo SAJ, Doty JB (2019). Epidemiologic and ecologic investigations of monkeypox, Likouala Department, Republic of the Congo, 2017. Emerg Infect Dis.

[73] Reynolds MG, Emerson GL, Pukuta E (2013). Detection of human monkeypox in the Republic of the Congo following intensive community education. Am J Trop Med Hyg.

[74] Learned LA, Reynolds MG, Wassa DW (2005). Extended interhuman transmission of monkeypox in a hospital community in the Republic of the Congo, 2003. Am J Trop Med Hyg.

[75] Sadeuh-Mba SA, Yonga MG, Els M (2019). Monkeypox virus phylogenetic similarities between a human case detected in Cameroon in 2018 and the 2017–2018 outbreak in Nigeria. Infect Genet Evol.

[76] Tchokoteu PF, Kago I, Tetanye E, Ndoumbe P, Pignon D, Mbede J (1991). [Variola or a severe case of varicella? A case of human variola due to monkeypox virus in a child from the Cameroon]. Ann Soc Belg Med Trop.

[77] Merouze F, Lesoin JJ (1983). [Monkeypox: second human case observed in Ivory Coast (rural health sector of Daloa]. Méd Trop (Mars).

[78] Breman JG, Nakano JH, Coffi E, Godfrey H, Gautun JC (1977). Human poxvirus disease after smallpox eradication. Am J Trop Med Hyg.

[79] Del Giudice P, Fribourg A, Roudiere L, Gillon J, Decoppet A, Reverte M (2023). Familial monkeypox virus infection involving 2 young children. Emerg Infect Dis.

[80] Vallée A, Chatelain A, Carbonnel M (2023). Monkeypox virus infection in 18-year-old woman after sexual intercourse, France, September 2022. Emerg Infect Dis.

[81] Antonello VS, Cornelio PE, Dallé J (2023). Disseminated neonatal monkeypox virus infection: case report in Brazil. Pediatr Infect Dis J.

[82] Meyer A, Esposito JJ, Gras F, Kolakowski T, Fatras M, Muller G (1991). [First appearance of monkey pox in human beings in Gabon]. Méd Trop (Mars).

[83] Foster SO, Brink EW, Hutchins DL (1972). Human monkeypox. Bull World Health Organ.

[84] Tutu van Furth AM, van der Kuip M, van Els AL (2022). Paediatric monkeypox patient with unknown source of infection, the Netherlands, June 2022. Euro Surveill.

[85] Reynolds MG, Wauquier N, Li Y (2019). Human monkeypox in Sierra Leone after 44-year absence of reported cases. Emerg Infect Dis.

[86] Formenty P, Muntasir MO, Damon I (2010). Human monkeypox outbreak caused by novel virus belonging to Congo Basin clade, Sudan, 2005. Emerg Infect Dis.

[87] Vaughan AM, Cenciarelli O, Colombe S (2022). A large multi-country outbreak of monkeypox across 41 countries in the WHO European Region, 7 March to 23 August 2022. Euro Surveill.

[88] Breman JG, Kalisa-Ruti, Steniowski MV, Zanotto E, Gromyko AI, Arita I (1980). Human monkeypox, 1970–79. Bull World Health Organ.

[89] UK Health Security Agency (2023). Mpox (monkeypox): background information. https://www.gov.uk/guidance/monkeypox.

[90] WHO (2023). Mpox (Monkeypox) outbreak toolbox. https://www.who.int/emergencies/outbreak-toolkit/disease-outbreak-toolboxes/mpox-outbreak-toolbox.

[91] Mitjà O, Ogoina D, Titanji BK (2023). Monkeypox. Lancet.

[92] Palich R, Burrel S, Monsel G (2023). Viral loads in clinical samples of men with monkeypox virus infection: a French case series. Lancet Infect Dis.

[93] Hoxha A, Kerr SM, Laurenson-Schafer H (2023). Mpox in children and adolescents during multicountry outbreak, 2022–2023. Emerg Infect Dis.

[94] WHO (2022).

[95] Bunge EM, Hoet B, Chen L (2022). The changing epidemiology of human monkeypox—a potential threat? A systematic review. PLoS Negl Trop Dis.

[96] Ministerio da Saude (2023). Boletim epidemiológico de monkeypox n° 22 (COE). https://www.gov.br/saude/pt-br/centrais-de-conteudo/publicacoes/boletins/epidemiologicos/variola-dos-macacos/boletim-epidemiologico-de-monkeypox-no-22-coe/view.

[97] Fox T, Gould S, Princy N, Rowland T, Lutje V, Kuehn R (2023). Therapeutics for treating mpox in humans. Cochrane Database Syst Rev.

[98] Shearer JD, Siemann L, Gerkovich M, House RV (2005). Biological activity of an intravenous preparation of human vaccinia immune globulin in mouse models of vaccinia virus infection. Antimicrob Agents Chemother.

[99] Ahmed SF, Sohail MS, Quadeer AA, McKay MR (2022). Vaccinia-virus-based vaccines are expected to elicit highly cross-reactive immunity to the 2022 monkeypox virus. Viruses.

[100] Thet AK, Kelly PJ, Kasule SN (2023). The use of vaccinia immune globulin in the treatment of severe mpox. virus infection in human immunodeficiency virus/AIDS. Clin Infect Dis.

[101] Ladhani SN, Dowell AC, Jones S (2023). Early evaluation of the safety, reactogenicity, and immune response after a single dose of modified vaccinia Ankara-Bavaria Nordic vaccine against mpox in children: a national outbreak response. Lancet Infect Dis.

[102] Islam M, Ahammed T, Ahmed Noor ST (2022). An estimation of five-decade long monkeypox case fatality rate: systematic review and meta-analysis. J Pure Appl Microbiol.

[103] Turner RM, Bird SM, Higgins JP (2013). The impact of study size on meta-analyses: examination of underpowered studies in Cochrane reviews. PLoS One.

[104] UK Government (2023). Foreign travel advice: Democratic Republic of the Congo. https://www.gov.uk/foreign-travel-advice/democratic-republic-of-the-congo.

[105] McCollum AM, Nakazawa Y, Ndongala GM (2015). Case report: human monkeypox in the Kivus, a conflict region of the Democratic Republic of the Congo. Am J Trop Med Hyg.

[106] UNHCR (2013). Emergency response for the situation in the eastern Democratic Republic of the Congo. https://www.unhcr.org/in/media/emergency-response-situation-eastern-democratic-republic-congo-march-2013.

[107] Durski KN, McCollum AM, Nakazawa Y (2018). Emergence of monkeypox—west and central Africa, 1970–2017. MMWR Morb Mortal Wkly Rep.

[108] McMullen CL, Mulembekani P, Hoff NA (2015). Human monkeypox transmission dynamics thirty years after smallpox eradication in the Sankuru district, Democratic Republic of Congo. Am J Trop Med Hyg.

[109] Hoff N, Ilunga B, Shongo R, Muyembe J, Mossoko M, Okitolonda E (2014). Human monkeypox disease surveillance and time trends in the Democratic Republic of Congo, 2001–2013. Am J Trop Med Hyg.

[110] Reynolds MG, Damon IK (2012). Outbreaks of human monkeypox after cessation of smallpox vaccination. Trends Microbiol.

[111] Shomuyiwa DO, Manirambona E (2023). Monkeypox virus declared as a global health emergency: what next for Africa's preparedness?. Travel Med Infect Dis.

[112] WHO (2022).

