# Supplementary material for: Paediatric, maternal, and congenital mpox: a systematic review and meta-analysis
Source: Lancet Glob Health. 2024 Feb 21;12(4):e572–88. doi: 10.1016/S2214-109X(23)00607-1 (PMC11519316; doi:10.1016/S2214-109X(23)00607-1)
Supplement: French translation of the abstract [file mmc1.pdf]

THE LANCET
Global Health

Supplementary appendix 1

This translation in French was submitted by the authors and we reproduce it as supplied. It has not been peer reviewed. *The Lancet*'s editorial processes have only been applied to the original in English, which should serve as reference for this manuscript.

Cette traduction en français a été proposée par les auteurs et nous l'avons reproduite telle quelle. Elle n'a pas été examinée par des pairs. Les processus éditoriaux du *Lancet* n'ont été appliqués qu'à l'original en anglais et c'est cette version qui doit servir de référence pour ce manuscrit.

Supplement to: Sanchez Clemente N, Coles C, Paixao ES, et al. Paediatric, maternal, and congenital mpox: a systematic review and meta-analysis. *Lancet Glob Health* 2024; published online Feb 21. https://doi.org/10.1016/S2214-109X(23)00607-1.

Résumé (Français):

Introduction: Bien que la mpox ait été détectée dans les populations pédiatriques d'Afrique centrale et de l'Ouest depuis des décennies, il n'existe pas de synthèse des données sur la mpox pédiatrique, maternelle et congénitale, ni sur l'utilisation des vaccins et des thérapies dans ces groupes. Une revue systématique est donc indiquée pour établir le programme de recherche.

Méthodes : Nous avons procédé à un examen systématique et à une méta-analyse (PROSPERO CRD420223336648), en recherchant des articles dans les bases de données EMBASE, Global Health, Medline, CINAHL, Web of Science, SCOPUS, Scielo et OMS. Nous avons inclus des essais cliniques et des études d'observation chez l'homme et exclu les revues, les commentaires et la littérature grise. Une estimation groupée du taux de létalité pédiatrique a été obtenue à l'aide d'une méta-analyse à effets aléatoires.

Résultats : Sur les 61 études, 53 ont fait état de résultats pédiatriques (n=2123 cas), 7 ont fait état de résultats maternels/congénitaux (n=32 cas), 2 ont fait état de la sécurité du vaccin (n=28 receveurs) et 3 ont fait état d'une transmission pendant l'allaitement (3 études, 4 cas). Bien qu'un sous-ensemble de 7 études d'observation (21 enfants et 12 femmes enceintes) ait fait état d'un traitement sans incident avec le tecovirimat, aucun essai randomisé n'a fait état de la sécurité et/ou de l'efficacité de l'un ou l'autre des agents thérapeutiques. Chez les enfants, les caractéristiques cliniques les plus fréquentes étaient l'éruption cutanée (86/86 ; 100%), la fièvre (63/86 ; 73%) et la lymphadénopathie (40/86 ; 47%). Parmi les femmes enceintes, une éruption cutanée a été signalée chez 23/23 (100 %) ; la fièvre et la lymphadénopathie étaient moins fréquentes (6/23 ; 26 % et 3/23 ; 13 %, respectivement). La plupart des complications pédiatriques (12/20 ; 60%) étaient dues à des infections bactériennes secondaires. Le taux de létalité pédiatrique regroupé était de 11 % (IC à 95 % : 4-20 %), I^=75 %. Les données relatives à 12 femmes enceintes ont montré que la moitié d'entre elles se sont soldées par une mort fœtale. La recherche sur la sécurité des vaccins et des immunoglobulines reste limitée pour les enfants et inexistante pour les femmes enceintes.

Interprétation : Notre étude met en évidence les lacunes critiques dans les connaissances sur l'épidémiologie, la prévention et le traitement de la mpox chez les enfants et les femmes enceintes, en particulier ceux qui résident dans des pays endémiques. Un financement accru, une collaboration internationale et une recherche équitable sont nécessaires pour élaborer des stratégies de contrôle de la mpox adaptées aux communautés à risque dans les pays endémiques.
