# Supplementary material for: Paediatric, maternal, and congenital mpox: a systematic review and meta-analysis
Source: Lancet Glob Health. 2024 Feb 21;12(4):e572–88. doi: 10.1016/S2214-109X(23)00607-1 (PMC11519316; doi:10.1016/S2214-109X(23)00607-1)
Supplement: Spanish translation of the abstract [file mmc2.pdf]

THE LANCET
Global Health

Supplementary appendix 2

This translation in Spanish was submitted by the authors and we reproduce it as supplied. It has not been peer reviewed. *The Lancet*'s editorial processes have only been applied to the original in English, which should serve as reference for this manuscript.

Los autores nos proporcionaron esta traducción al español y la reproducimos tal como nos fue entregada. No la hemos revisado. Los procesos editoriales de *The Lancet* se han aplicado únicamente al original en inglés, que debe servir de referencia para este manuscrito.

Supplement to: Sanchez Clemente N, Coles C, Paixao ES, et al. Paediatric, maternal, and congenital mpox: a systematic review and meta-analysis. *Lancet Glob Health* 2024; published online Feb 21. https://doi.org/10.1016/S2214-109X(23)00607-1.

Resumen (español):

Introducción: Aunque el mpox se ha detectado en poblaciones pediátricas de África Central y Occidental durante décadas, falta una síntesis de la evidencia sobre el mpox pediátrico, materno y congénito, y el uso de vacunas y terapéutica en estos grupos. Por lo tanto, está indicada una revisión sistemática para establecer la agenda de investigación.

Métodos: Se realizó una revisión sistemática y metaanálisis (PROSPERO CRD420223336648), buscando artículos en las bases de datos EMBASE, Global Health, Medline, CINAHL, Web of Science, SCOPUS, Scielo y OMS. Se incluyeron ensayos clínicos y estudios observacionales en humanos y se excluyeron revisiones, comentarios y literatura gris. Se obtuvo una estimación conjunta de la tasa de letalidad pediátrica mediante un metanálisis de efectos aleatorios.

Resultados: De los 61 estudios, 53 notificaron resultados pediátricos (n=2123 casos), 7 notificaron resultados maternos/congénitos (n=32 casos), 2 notificaron la seguridad de la vacuna (n=28 casos) y 3 notificaron la transmisión durante la lactancia (3 estudios, 4 casos). Mientras que un subconjunto de 7 estudios observacionales (21 niños y 12 embarazadas) informaron de un tratamiento sin complicaciones con tecovirimat, no hubo ensayos clínicos que informaran de la seguridad y/o eficacia de ningún agente terapéutico. Entre los niños, las características clínicas más frecuentes fueron erupción cutánea (86/86; 100%), fiebre (63/86; 73%) y linfadenopatía (40/86; 47%). Entre las embarazadas, 23/23 (100%) presentaron exantema; la fiebre y las linfadenopatías fueron menos frecuentes (6/23; 26% y 3/23; 13%, respectivamente). La mayoría de las complicaciones pediátricas (12/20; 60%) se debieron a infecciones bacterianas secundarias. La tasa de letalidad pediátrica combinada fue del 11% (IC 95%: 4-20%), I^=75%. Los datos de 12 embarazos mostraron que la mitad resultaron en muerte fetal. La investigación sobre la seguridad de las vacunas y las inmunoglobulinas sigue siendo limitada en el caso de los niños y ausente en el de las embarazadas.

Interpretación: Nuestra revisión destaca las lagunas críticas de conocimiento en la epidemiología, prevención y tratamiento del mpox en niños y embarazadas, especialmente en aquellos que residen en países endémicos. Es necesario aumentar la financiación, la colaboración internacional y la investigación equitativa para fundamentar las estrategias de control del mpox adaptadas a las comunidades de riesgo de los países endémicos.
