# Supplementary material for: Paediatric, maternal, and congenital mpox: a systematic review and meta-analysis
Source: Lancet Glob Health. 2024 Feb 21;12(4):e572–88. doi: 10.1016/S2214-109X(23)00607-1 (PMC11519316; doi:10.1016/S2214-109X(23)00607-1)
Supplement: Portuguese translation of the abstract [file mmc3.pdf]

THE LANCET
Global Health

Supplementary appendix 3

This translation in Portuguese was submitted by the authors and we reproduce it as supplied. It has not been peer reviewed. *The Lancet*'s editorial processes have only been applied to the original in English, which should serve as reference for this manuscript.

Esta tradução em português foi submetida pelos autores e nós não fizemos quaisquer alterações. Esta versão não foi revista por pares. O processo editorial do The Lancet só foi aplicado à versão original em inglês, que deve servir como referência para este artigo.

Supplement to: Sanchez Clemente N, Coles C, Paixao ES, et al. Paediatric, maternal, and congenital mpox: a systematic review and meta-analysis. *Lancet Glob Health* 2024; published online Feb 21. https://doi.org/10.1016/S2214-109X(23)00607-1.

Resumo (Português):

Introdução: Embora a mpox tenha sido detectada em populações pediátricas na África Central e Ocidental há décadas, faltam evidências sobre a mpox pediátrica, materna e congénita, e a utilização de vacinas e terapias nestes grupos. Por conseguinte, é indicada uma revisão sistemática para definir as prioridades de pesquisa.

Métodos: Realizámos uma revisão sistemática e uma meta-análise (PROSPERO CRD420223336648), pesquisando artigos nas bases de dados EMBASE, Global Health, Medline, CINAHL, Web of Science, SCOPUS, Scielo e OMS. Incluímos ensaios clínicos e estudos observacionais em seres humanos e excluímos revisões, comentários e literatura cinzenta. Obteve-se uma estimativa conjunta do rácio de mortalidade pediátrica utilizando uma meta-análise de efeitos aleatórios.

Resultados: Dos 61 estudos, 53 relataram resultados pediátricos (n=2123 casos), 7 relataram resultados maternos/congénitos (n=32 casos), 2 relataram a segurança da vacina (n=28 casos) e 3 relataram a transmissão durante a amamentação (3 estudos, 4 casos). Embora um subconjunto de 7 estudos observacionais (21 crianças e 12 grávidas) tenha relatado um tratamento sem intercorrências com tecovirimat, não houve ensaios clínicos que relatassem a segurança e/ou eficácia de qualquer agente terapêutico. Entre as crianças, as características clínicas mais comuns incluíram erupção cutânea (86/86; 100%), febre (63/86; 73%) e linfadenopatia (40/86; 47%). Entre os casos na gravidez, a erupção cutânea foi registada em 23/23 (100%); a febre e a linfadenopatia foram menos comuns (6/23; 26% e 3/23; 13%, respetivamente). A maioria das complicações pediátricas (12/20; 60%) teve origem em infecções bacterianas secundárias. A taxa de mortalidade pediátrica combinada foi de 11% (95% CI 4-20%), I^=75%. Os dados de 12 gravidezes mostraram que metade resultou em morte fetal. Dados sobre a segurança das vacinas e das imunoglobulinas continua a ser limitada no caso das crianças e inexistente no caso das grávidas.

Interpretação: A nossa revisão destaca lacunas críticas de conhecimento na epidemiologia, prevenção e tratamento da mpox em crianças e grávidas, especialmente nas que residem em países endémicos. É necessário aumentar o financiamento, a colaboração internacional e a investigação equitativa para informar as estratégias de controlo da mpox adaptadas às comunidades em risco nos países endémicos.
