# Supplementary material for: Paediatric, maternal, and congenital mpox: a systematic review and meta-analysis
Source: Lancet Glob Health. 2024 Feb 21;12(4):e572–88. doi: 10.1016/S2214-109X(23)00607-1 (PMC11519316; doi:10.1016/S2214-109X(23)00607-1)
Supplement: Supplementary appendix 4 [file mmc4.pdf]

THE LANCET
Global Health

Supplementary appendix 4

This appendix formed part of the original submission and has been peer reviewed. We post it as supplied by the authors.

Supplement to: Sanchez Clemente N, Coles C, Paixao ES, et al. Paediatric, maternal, and congenital mpox: a systematic review and meta-analysis. *Lancet Glob Health* 2024; published online Feb 21. https://doi.org/10.1016/S2214-109X(23)00607-1.

Appendix

Table 1. PICOS Criteria

**Population of interest**	Pregnant women, neonates and children ≤18 years in all countries in all time periods
**Intervention/Exposure of interest**	Exposure: Mpox virus infection<br>Intervention: The use of third-generation vaccines or targeted antivirals or immune therapies in at least one case of mpox in the population of interest
**Comparator**	N/A
**Outcomes of interest**	-Preterm labour, early or late pregnancy loss, maternal hospitalization, requirement of maternal intensive care, maternal mortality<br>-Mother-to-child transmission of monkeypox during pregnancy<br>- Mother-to-child transmission of monkeypox during breastfeeding<br>-Prematurity, small for gestational age, low birthweight, microcephaly, congenital anomalies, requirement of neonatal intensive care, neonatal death<br>-Paediatric hospital admission, requirement for paediatric intensive care<br>-Safety and adverse events of smallpox vaccine in pregnancy and in neonatal and paediatric age groups (using GAIA criteria[1]).<br>-Efficacy of smallpox vaccine in preventing maternal, congenital, neonatal and paediatric monkeypox infection<br>-Safety and efficacy of the use of targeted antivirals or immune therapies in the treatment of monkeypox in pregnancy to reduce the risk of vertical transmission<br>-Safety and efficacy of the use of targeted antivirals or immune therapies in the treatment of monkeypox in neonates, infants and children to reduce morbidity, mortality or viral load
**Study designs**	All study designs reporting primary data on paediatric, maternal and congenital mpox in humans in any setting and any country in any time period

Paediatric/Maternal MPOX case definitions

(Adapted from: World Health Organization (21 May 2022). Disease Outbreak News; Multi-country monkeypox outbreak in non-endemic countries.)

Suspected case:

A child/young person 0-18 years old/pregnant person presenting in a monkeypox endemic or non-endemic country[2] with an unexplained acute rash

AND

One or more of the following signs or symptoms:

- Headache
- Acute onset of fever (>38.5°C),
- Lymphadenopathy (swollen lymph nodes)
- Myalgia (muscle and body aches)
- Back pain
- Asthenia (profound weakness)

AND

For which the following common causes of acute rash do not explain the clinical picture: varicella zoster, herpes zoster, measles, Zika, dengue, chikungunya, herpes simplex, bacterial skin infections, disseminated g*onococcus* infection, primary or secondary syphilis, chancroid, lymphogranuloma venereum, granuloma inguinale, molluscum contagiosum, allergic reaction (e.g., to plants); and any other locally relevant common causes of papular or vesicular rash.

N.B. It is not necessary to obtain negative laboratory results for listed common causes of rash illness in order to classify a case as suspected.

[2] Monkeypox endemic countries are: Benin, Cameroon, the Central African Republic, the Democratic Republic of the Congo, Gabon, Ghana (identified in animals only), Côte d'Ivoire, Liberia, Nigeria, the Republic of the Congo, and Sierra Leone. Benin and South Sudan have documented importations in the past. Countries currently reporting cases of the West African clade are Cameroon and Nigeria. With this case definition, all countries except these four should report new cases of monkeypox as part of the current multi-country outbreak.

Probable case:

A person meeting the case definition for a suspected case

AND

One or more of the following:

- Has an epidemiological link (face-to-face exposure, including health workers without eye and respiratory protection); direct physical contact with skin or skin lesions, including sexual contact; or contact with contaminated materials such as clothing, bedding or utensils to a probable or confirmed case of monkeypox in the 21 days before symptom onset
- Reported travel history to a monkeypox endemic country[2] in the 21 days before symptom onset
- Has had multiple or anonymous sexual partners in the 21 days before symptom onset
- Has a positive result of an *orthopoxvirus* serological assay, in the absence of smallpox vaccination or other known exposure to orthopoxviruses
- Is hospitalized due to the illness

Confirmed case:

A case meeting the definition of either a suspected or probable case and is laboratory confirmed for monkeypox virus by detection of unique sequences of viral DNA either by real-time polymerase chain reaction (PCR) and/or sequencing.

Vertical MPX transmission:

Defined as the presence of laboratory-confirmed analysis on fetal or neonatal sample (amniotic fluid or cord blood) or the presence of clinical manifestations suggestive of monkeypox infection.

Note: The authors acknowledge the variability in clinical presentation of mpox cases, particularly the differences between the clinical presentation of cases in Clade IIa/b (primarily genital and perianal cutaneous lesions with minimal systemic features) and Clade I (widespread cutaneous lesions with more frequent appearance of systemic features such as fever, lymphadenopathy and ocular involvement). The WHO case definitions were designed to be inclusive to capture clinical features of all the clades therefore the authors and reviewers considered it was reasonable to use the WHO case definition that was drafted for the 2022/2023 worldwide outbreak.

Mpox endemic countries with data in the SR: Cameroon, Central African Republic (CAR), Côte d'Ivoire, Democratic Republic of Congo (DRC), Gabon, Liberia, Nigeria, The Republic of the Congo, Sierra Leone, Sudan (pre-2011 split).
Mpox non-endemic countries with data in the SR: Brazil, France, The Netherlands, Spain, The United Kingdom of Great Britain and Northern Ireland, The United States of America.

Case Fatality Ratio (CFR) Definition:
Case fatality rate, also called case fatality risk or case fatality ratio, in epidemiology, the proportion of people who die from a specified disease among all individuals diagnosed with the disease over a certain period of time.

From: Encyclopedia Britannica. https://www.britannica.com/science/case-fatality-rate

Table 2. Study Quality Assessments according to MURAD Criteria

Author	Year	1. Does the patient(s) represent(s) the whole experience of the investigator (centre) or is the selection method unclear to the extent that other patients with similar presentation may not have been reported?	2. Was the exposure adequately ascertained?	3. Was the outcome adequately ascertained?	4. Were other alternative causes that may explain the observation ruled out?	5. Was there a challenge/re-challenge phenomenon?	6. Was there a dose–response effect?	7. Was follow-up long enough for outcome to occur?	8. Is the case(s) described with sufficient details to allow other investigators to replicate the research or to allow practitioners make inferences related to their own practice?	Overall score
Pittman	2023	Y	Y	Y	Y	N	N	Y	Y	Good
Ditta	2023	Y	N	Y	Y	N	Y	Y	Y	Good
Antonello	2023	Y	Y	Y	Y	N	N	Y	Y	Good
Del Giudice	2023	Y	Y	Y	Y	N	N	Y	Y	Good
Sampson	2023	Y	Y	Y	Y	N	N	Y	Y	Good
Oakley	2023	Y	Y	N	N	N	N	Y	Y	Fair
Minhaj	2022	Y	Y	Y	Y	N	N	Y	Y	Good
Ladhani	2022	Y	Y	Y	N	N	N	Y	Y	Good
Alonso Cadenas	2022	Y	Y	Y	N	N	N	Y	Y	Good
Vallee	2022	Y	Y	Y	Y	N	N	Y	Y	Good
Roguera Sopena	2022	Y	Y	Y	Y	N	N	Y	Y	Good
Hennessee	2022	Y	N	Y	Y	N	N	Y	Y	Fair
Fuente	2022	Y	N	Y	Y	N	N	N	Y	Fair
van Furth	2022	Y	N	N	Y	N	N	N	Y	Fair
Aguilera Alonso	2022	Y	N	Y	Y	N	N	Y	Y	Fair
Besombes	2022	Y	Y	Y	Y	N	N	Y	N	Fair
Ramnarayan	2022	Y	Y	Y	Y	N	N	Y	Y	Good
Stanek	2022	Y	Y	Y	Y	N	N	Y	Y	Good
Vaughan	2022	Y	Y	Y	N	N	N	N	Y	Fair
Adler	2022	Y	Y	Y	Y	N	N	Y	Y	Good
Whitehouse	2021	Y	Y	N	Y	N	N	N	Y	Fair
Hobson	2021	Y	Y	Y	Y	N	N	Y	Y	Good
Ogoina	2020	Y	N	Y	N	N	N	Y	N	Fair
Eltvedt	2020	Y	N	Y	N	N	N	Y	N	Fair
Yinka-Ogunleye	2019	Y	Y	Y	Y	N	N	Y	Y	Good
Sadeuh-Mba	2019	N	Y	Y	N	N	N	N	N	Poor
Reynolds	2019	Y	Y	Y	N	N	N	Y	N	Fair
Ogoina	2019	Y	Y	N	Y	N	N	N	N	Fair
Doshi	2019	Y	Y	Y	Y	N	N	Y	Y	Good
Besombes	2019	Y	Y	Y	Y	N	N	Y	Y	Good
Kalthan	2018	Y	Y	Y	Y	N	N	Y	Y	Good
Nakoune	2017	Y	Y	Y	Y	N	N	Y	Y	Good

Mbala	2017	Y	Y	Y	Y	N	N	Y	Y	Good
Kalthan	2016	Y	Y	Y	N	N	N	Y	Y	Fair
Johnston	2015	Y	Y	N	Y	N	N	N	N	Fair
Reynolds	2013	Y	Y	N	Y	N	N	N	N	Fair
Formenty	2010	Y	Y	Y	Y	N	N	Y	Y	Good
Learned	2005	Y	Y	Y	Y	N	N	Y	Y	Good
Sejvar	2004	Y	Y	Y	Y	N	N	Y	Y	Good
Anderson	2003	Y	Y	Y	N	N	N	N	Y	Fair
Meyer	2002	Y	Y	Y	Y	N	N	Y	Y	Good
Hutin	2001	Y	N	N	N	N	N	Y	N	Poor
CDC	1997	Y	Y	Y	Y	N	N	Y	Y	Good
Tchokoteu	1991	N	Y	Y	Y	N	N	Y	Y	Fair
Meyer	1991	N	Y	Y	Y	N	N	Y	Y	Fair
Herve	1989	N	Y	Y	N	N	N	Y	N	Fair
Jezek (Bull WHO)	1988	Y	Y	Y	Y	N	N	Y	Y	Good
Jezek (Trop Geog)	1987	Y	Y	Y	N	N	N	Y	Y	Fair
Jezek (JTMH)	1987	Y	Y	Y	Y	N	N	Y	Y	Good
Jezek (JID)	1987	Y	Y	Y	Y	N	N	Y	Y	Good
Jezek	1986	Y	Y	Y	N	N	N	Y	Y	Fair
Khodakevich	1985	N	N	Y	N	N	N	Y	N	Poor
Janseghers	1984	Y	Y	Y	N	N	N	Y	Y	Fair
Wa Mutombo	1983	Y	N	Y	Y	N	N	Y	Y	Fair
Merouze	1983	Y	N	Y	Y	N	N	Y	Y	Fair
Jezek	1983	N	Y	Y	N	N	N	Y	N	Fair
Breman	1980	Y	Y	Y	N	N	N	Y	Y	Fair
Breman	1977	Y	Y	Y	Y	N	N	Y	Y	Good
Ladnyj	1972	Y	Y	Y	N	N	N	Y	Y	Fair
Foster	1972	Y	Y	Y	N	N	N	Y	Y	Fair
Eke	1972	Y	Y	Y	Y	N	N	Y	Y	Good

*Based on number of YES replies: 0-2 = poor, 3-5 = fair, 6-8 = good

Figure 1. Age of Paediatric Monkeypox individual cases (n=96). Systematic Review of paediatric, maternal and congenital mpox, 2023.

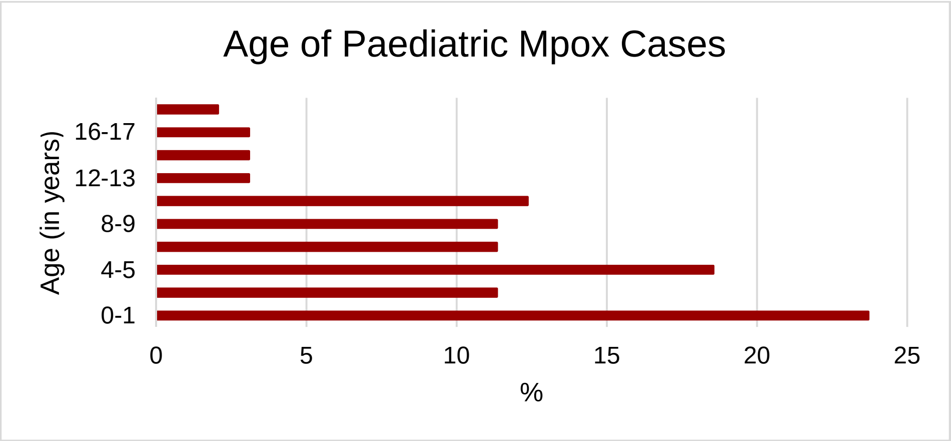

Table 3. Paediatric symptoms and signs stratified by country of origin (endemic or non-endemic for mpox)

Symptoms/signs	Overall (n=86)	% (95% CI)	Endemic (n=74)	% (95% CI)	Non-endemic (n=12)	% (95% CI)
Rash	86	100	74	100	12	100
Fever	63	73 (63-82)	62	84 (73-91)	1	8 (0-4)
Lymphadenopathy/adenitis	40	47 (36-58)	38	51 (57-79)	2	17 (2-48)
Dysphagia/Tonsillitis/pharyngitis	15	17 (10-27)	14	19 (16-37)	1	8 (0-4)
Conjunctivitis	9	10 (5-19)	7	9 (6-22)	2	17 (2-48)
Malaise/fatigue	7	8 (3-16)	7	9 (6-22)	0	0
Headache	6	7 (3-15)	6	8 (5-20)	0	0
Facial oedema	6	7 (3-15)	6	8 (5-20)	0	0
Vomiting/diarrhoea	5	6 (2-13)	4	5 (2-15)	1	8 (2-48)
Hepatosplenomegaly	4	5 (1-11)	4	5 (2-15)	0	0
Night sweats/rigors	4	5 (1-11)	4	5 (2-15)	0	0
Arthralgia/myalgia	3	3 (1-10)	3	4 (1-13)	0	0
Parotitis	3	3 (1-10)	3	4 (1-13)	0	0
Cough	2	2 (0-8)	2	3 (1-11)	0	0
Abdominal pain	1	1 (0-6)	1	1 (0-7)	0	0
Genital rash	1	1 (0-6)	0	0	1	8 (2.48)

Paediatric Supportive treatments:

Oral, parenteral or topical (ocular) antibiotics, intravenous fluids or parenteral nutrition, vitamins/minerals such as retinol, vitamin B and iron, oxygen, furosemide, promethazine (a sedating anti-histamine), maxilase (an anti-inflammatory drug), bronchodilators and inhaled and systemic steroids (in a case of encephalitis). Lorazepam and phenobarbitone were used in a separate case of encephalitis. Isoprinosine (an immunomodulatory drug) was used in four cases and moroxydine hydrochloride (an antiviral) in one case.

Paediatric Complications:

Secondary bacterial complications: Sepsis (2/20),[41] pneumonia (2/20),[21,43] otitis/mastoiditis (1/20),[45] preseptal cellulitis (1/20)[52] retropharyngeal abscess (1/20),[55] meningitis (1/20),[41] renal tract sepsis (1/20),[41] cellulitis (1/20) and lymphadenitis (2/20).[45,80]

Other complications: Seizures/encephalitis (2/20),[43,54] haemorrhagic complications including haematemesis (1/20)[79] and buccal bleeding secondary to liver failure and coagulopathy in (2/20).[79]

Respiratory complications: Tachypnoea (1/20),[73] respiratory distress syndrome (2/20),[42,78] pulmonary oedema (2/20),[12] respiratory failure (1/20)[7] and hypoxaemia requiring ventilation (2/20).[69,78]

Table 4. Details of all included maternal, breastfeeding and vaccine studies in pregnant and paediatric populations. Systematic Review of paediatric, maternal and congenital mpox, 2023.

Vaccine studies						
Author, year	Study type	Country	n	Age of children	Vaccine type/Study period	Findings
Ladhani, 2022	Observational study	UK	21	<12 years	MVA-BN (IMVANEX)* May-Jul 2022	21 children < 12 years were vaccinated. Across different school outbreaks: 7 children age 2-3 years, 4 children age 4-5 years, 10 children age 5-11 years.
Minhaj, 2022	Case series	USA	8	0-14	MVA-BN (JYNNEOS)*/ VIGIV as PEP May-Sept 2022	Seven children (aged 0–14 years) received PEP with MVA-BN. No side effects reported. In a separate outbreak, one neonate received vaccinia immune globulin intravenous (VIGIV) under a single patient emergency Investigational New Drug application due to suspected congenital mpox. No reported side effects. All children in the study subsequently found not to have mpox.

Search terms
Medline, EMBASE and Global Health:

1. Monkeypox/ or Monkeypox virus/ or mpox

2. (monkeypox or monkey pox).af.

3. 1 or 2

4. pregnan*.tw,kf

5. pregnancy/ or pregnancy outcome/ or pregnancy complications, infectious/

6. obstetric*.tw,kf

7. obstetrics/

8. (maternal or mother*).tw,kf

9. Mothers/

10. (f?etal or f?etus*).tw,kf

11. fetus/

12. (neonat* or newborn* or new-born*).tw,kf

13. (infant* or infancy).tw,kf

14. exp infant/

15. child*.tw,kf

16. exp child/

17. p?ediatric*.tw,kf

18. exp pediatrics/

19. congenital*.tw,kf

20. (breastfe?d* or breast fe?d*).tw,kf

21. (vertical* adj3 transmi*).tw,kf

22. Infectious Disease Transmission, Vertical/

23. or/4-22

24. 3 and 23

1. vaccin*.tw,kf.

2. vaccines/ or viral vaccines/ or Smallpox Vaccine/ or vaccination/

3. (tecovirimat or TPOXX or ST-246).af

4. (antiviral* or anti-viral*).tw,kf

5. Antiviral Agents/

6. or/1-5

7. (monkeypox or monkey pox).af

8. Monkeypox/ or Monkeypox virus/

9. 7 or 8

10. pregnan*.tw,kf.

11. pregnancy/ or pregnancy outcome/ or pregnancy complications/

12. (maternal or mother*).tw,kf.

13. Mothers/

14. (neonat* or newborn* or new-born*).tw,kf.

15. (infant* or infancy).tw,kf.

16. exp infant/

17. child*.tw,kf.

18. exp child/

19. p?ediatric*.tw,kf.

20. exp pediatrics/

21. congenital*.tw,kf.

22. (breastfe?d* or breast fe?d*).tw,kf.

23. Breast Feeding/

24. (vertical* adj3 transmi*).tw,kf.

25. Infectious Disease Transmission, Vertical/

26. or/10-25

27. 6 and 9 and 26

1. Smallpox Vaccine/

2. (smallpox.tw,kf) and (vaccines/ or viral vaccines/ or vaccination/ or vaccin*.tw,kf)

3. (tecovirimat or TPOXX or ST-246).af.

4. or/1-3

5. pregnan*.tw,kf.

6. pregnancy/ or pregnancy outcome/ or pregnancy complications/

7. (neonat* or newborn* or new-born*).tw,kf.

8. (infant* or infancy).tw,kf.

9. exp infant/

10. child*.tw,kf.

11. exp child/

12. p?ediatric*.tw,kf.

13. exp pediatrics/

14. or/5-13

15. safe or safety.tw,kf

16. Safety/

17. (risk or risks or harm or harms).tw,kf.

18. risk factors/

19. (adverse adj2 (effect* or event* or outcome*)).tw,kf.

20. or/15-19

21. 4 and 14 and 20

CINAHL:

S21	S3 AND S20	**Expanders** - Apply equivalent subjects<br>**Search modes** - Boolean/Phrase
S20		S4 OR S5 OR S6 OR S7 OR S8 OR S9 OR S10 OR S11 OR S12 OR S13 OR S14 OR [illegible]
S19		"Infectious Disease Transmission, Vertical"
S18		vertical* AND transmi*
S17		breastfe?d* OR "breast fe?d*"
S16		congenital*
S15		p?ediatric*
S14		child*
S13		infant* OR infancy
S12		neonat* OR newborn* OR new-born*
S11		fetus
S10		f?etal OR f?etus*
S9		Mothers
S8		maternal OR mother*
S7		obstetrics
S6		obstetric*
S5		pregnancy OR pregnancy outcome OR "pregnancy complications"
S4		pregnan*
S3		S1 OR S2
S2		monkeypox OR "monkey pox"
S1		Monkeypox OR "Monkeypox virus"

SCIELO:

Id.	Search
#20	**Expression:** #2 AND # 19<br>**Filters:**
#19	**Expression:** #3 OR #4 OR #5 OR #6 OR #7 OR #8 OR #9 OR #10 OR #11 OR #12 OR #13 OR #14 C<br>**Filters:**
#18	**Expression:** #3 OR #4 OR #5 OR #6 OR #7 OR #8 OR #9 OR #10 OR #11 OR #12 OR #13 OR<br>**Filters:**
#17	**Expression:** vertical*<br>**Filters:**
#16	**Expression:** breastfe$d*<br>**Filters:**
#15	**Expression:** congenital*<br>**Filters:**
#14	**Expression:** p$ediatric*<br>**Filters:**
#13	**Expression:** child*<br>**Filters:**
#12	**Expression:** (infant*) OR (infancy)<br>**Filters:**
#11	**Expression:** (neonat*) OR (newborn*) OR (new-born*)<br>**Filters:**
#10	**Expression:** fetus<br>**Filters:**
#9	**Expression:** (f$etal) OR (f$etus*)<br>**Filters:**
#8	**Expression:** Mothers<br>**Filters:**
#7	**Expression:** (maternal) OR (mother*)<br>**Filters:**
#6	**Expression:** obstetrics<br>**Filters:**
#5	**Expression:** obstetric*<br>**Filters:**
#4	**Expression:** (pregnancy) OR ("pregnancy outcome") OR ("pregnancy complications, infectious")<br>**Filters:**
#3	**Expression:** pregnan*<br>**Filters:**
#2	**Expression:** ("Monkeypox virus") OR (monkeypox) OR ("monkey pox")<br>**Filters:**

#1 **Expression:** Monkeypox
Filters:

Scopus:

((TITLE-ABS-KEY (pregnan*)) OR (TITLE-ABS-KEY (pregnancy OR "pregnancy outcome" OR "pregnancy complications, infectious")) OR (TITLE-ABS-KEY (obstetric*)) OR (TITLE-ABS-KEY (obstetrics/)) OR (TITLE-ABS-KEY (maternal OR mother*)) OR (TITLE-ABS-KEY (mothers/)) OR (TITLE-ABS-KEY (f?etal OR f?etus*)) OR (TITLE-ABS-KEY (fetus/)) OR (TITLE-ABS-KEY (neonat* OR newborn* OR new-born*)) OR (TITLE-ABS-KEY (infant* OR infancy)) OR (TITLE-ABS-KEY (exp AND infant/)) OR (TITLE-ABS-KEY (child*)) OR (TITLE-ABS-KEY (exp AND child/)) OR (TITLE-ABS-KEY (p?ediatric*)) OR (TITLE-ABS-KEY (congenital*)) OR (TITLE-ABS-KEY (breastfe?d* OR "breast fe?d*")) OR (TITLE-ABS-KEY (vertical* W/3 transmi*)) OR (TITLE-ABS-KEY ("infectious disease transmission, vertical/"))) AND ((monkeypox OR "monkeypox virus") OR (TITLE-ABS-KEY (monkeypox OR "monkey pox")))

Table 4. PRISMA checklist:

Section and Topic	Item #	Checklist item	Location where item is reported
**TITLE**			
Title	1	Identify the report as a systematic review.	Pg. 1
**ABSTRACT**			
Abstract	2	See the PRISMA 2020 for Abstracts checklist.	Pg. 2
**INTRODUCTION**			
Rationale	3	Describe the rationale for the review in the context of existing knowledge.	Pg. 4
Objectives	4	Provide an explicit statement of the objective(s) or question(s) the review addresses.	Pg. 4
**METHODS**			
Eligibility criteria	5	Specify the inclusion and exclusion criteria for the review and how studies were grouped for the syntheses.	Pg. 5 and Appendix (PICOS)
Information sources	6	Specify all databases, registers, websites, organisations, reference lists and other sources searched or consulted to identify studies. Specify the date when each source was last searched or consulted.	Pg. 4 and Figure 1
Search strategy	7	Present the full search strategies for all databases, registers and websites, including any filters and limits used.	Appendix pgs. 7-9
Selection process	8	Specify the methods used to decide whether a study met the inclusion criteria of the review, including how many reviewers screened each record and each report retrieved, whether they worked independently, and if applicable, details of automation tools used in the process.	Pg. 5
Data collection	9	Specify the methods used to collect data from reports, including how many	Pg. 5

Section and Topic	Item #	Checklist item	Location where item is reported
process		reviewers collected data from each report, whether they worked independently, any processes for obtaining or confirming data from study investigators, and if applicable, details of automation tools used in the process.	
Data items	10a	List and define all outcomes for which data were sought. Specify whether all results that were compatible with each outcome domain in each study were sought (e.g. for all measures, time points, analyses), and if not, the methods used to decide which results to collect.	Pg. 5 and Appendix pg. 1 (PICOS)
	10b	List and define all other variables for which data were sought (e.g. participant and intervention characteristics, funding sources). Describe any assumptions made about any missing or unclear information.	Pg. 5 and Appendix pg. 1 (PICOS)
Study risk of bias assessment	11	Specify the methods used to assess risk of bias in the included studies, including details of the tool(s) used, how many reviewers assessed each study and whether they worked independently, and if applicable, details of automation tools used in the process.	Pg. 5-6
Effect measures	12	Specify for each outcome the effect measure(s) (e.g. risk ratio, mean difference) used in the synthesis or presentation of results.	Pg. 5-6
Synthesis methods	13a	Describe the processes used to decide which studies were eligible for each synthesis (e.g. tabulating the study intervention characteristics and comparing against the planned groups for each synthesis (item #5)).	Pg. 5
	13b	Describe any methods required to prepare the data for presentation or synthesis, such as handling of missing summary statistics, or data conversions.	Pg. 5-6
	13c	Describe any methods used to tabulate or visually display results of individual studies and syntheses.	Pg. 5-6
	13d	Describe any methods used to synthesize results and provide a rationale for the choice(s). If meta-analysis was performed, describe the model(s), method(s) to identify the presence and extent of statistical heterogeneity, and software package(s) used.	Pg. 5-6
	13e	Describe any methods used to explore possible causes of heterogeneity among study results (e.g. subgroup analysis, meta-regression).	Pg. 6
	13f	Describe any sensitivity analyses conducted to assess robustness of the synthesized results.	N/A
Reporting bias assessment	14	Describe any methods used to assess risk of bias due to missing results in a synthesis (arising from reporting biases).	Pg. 6
Certainty assessment	15	Describe any methods used to assess certainty (or confidence) in the body of evidence for an outcome.	Pgs. 5-6
**RESULTS**			
Study selection	16a	Describe the results of the search and selection process, from the number of records identified in the search to the number of studies included in the review, ideally using a flow diagram.	Figure 1 and pg. 6
	16b	Cite studies that might appear to meet the inclusion criteria, but which were excluded, and explain why they were excluded.	Figure 1
Study characteristics	17	Cite each included study and present its characteristics.	Page 6
Risk of bias in studies	18	Present assessments of risk of bias for each included study.	Table 4
Results of individual studies	19	For all outcomes, present, for each study: (a) summary statistics for each group (where appropriate) and (b) an effect estimate and its precision (e.g. confidence/credible interval), ideally using structured tables or plots.	Figures 4 and 5 and Pgs. 6-8
Results of syntheses	20a	For each synthesis, briefly summarise the characteristics and risk of bias among contributing studies.	Pgs. 6-8
	20b	Present results of all statistical syntheses conducted. If meta-analysis was	Figure 5

Section and Topic	Item #	Checklist item	Location where item is reported
		done, present for each the summary estimate and its precision (e.g. confidence/credible interval) and measures of statistical heterogeneity. If comparing groups, describe the direction of the effect.	
	20c	Present results of all investigations of possible causes of heterogeneity among study results.	Figure 5
	20d	Present results of all sensitivity analyses conducted to assess the robustness of the synthesized results.	N/A
Reporting biases	21	Present assessments of risk of bias due to missing results (arising from reporting biases) for each synthesis assessed.	Table 4
Certainty of evidence	22	Present assessments of certainty (or confidence) in the body of evidence for each outcome assessed.	Pgs. 6-9
**DISCUSSION**			
Discussion	23a	Provide a general interpretation of the results in the context of other evidence.	Pg. 9
	23b	Discuss any limitations of the evidence included in the review.	Pg. 10
	23c	Discuss any limitations of the review processes used.	Pg. 10-11
	23d	Discuss implications of the results for practice, policy, and future research.	Pg. 11
**OTHER INFORMATION**			
Registration and protocol	24a	Provide registration information for the review, including register name and registration number, or state that the review was not registered.	Pg. 5
	24b	Indicate where the review protocol can be accessed, or state that a protocol was not prepared.	Pg. 5
	24c	Describe and explain any amendments to information provided at registration or in the protocol.	N/A
Support	25	Describe sources of financial or non-financial support for the review, and the role of the funders or sponsors in the review.	Pg. 2
Competing interests	26	Declare any competing interests of review authors.	Pg. 12
Availability of data, code and other materials	27	Report which of the following are publicly available and where they can be found: template data collection forms; data extracted from included studies; data used for all analyses; analytic code; any other materials used in the review.	Pg. 12

From: Page MJ, McKenzie JE, Bossuyt PM, Boutron I, Hoffmann TC, Mulrow CD, et al. The PRISMA 2020 statement: an updated guideline for reporting systematic reviews. BMJ 2021;372:n71. doi: 10.1136/bmj.n71

For more information, visit: http://www.prisma-statement.org/
